# Oncolytic virotherapy for cancer: biological mechanisms, clinical advances, and future perspectives

**DOI:** 10.1007/s10238-026-02311-3

**Published:** 2026-10-16

**Authors:** Esraa M. Halawa, Sherihan G. AbdelHamid, Mahmoud ElHefnawi

**Affiliations:** 1 Department of Botany and Microbiology, Faculty of Science, Cairo University, Giza, 12613 Egypt; 2 Department of Biochemistry and Molecular Biology, Faculty of Pharmacy, Ain Shams University, Cairo, 11566 Egypt; 3 Biomedical Informatics and Chemoinformatics Group, Informatics and Systems Department, National Research Centre, Tahrir Street, Cairo, 12311 Egypt

**Keywords:** Oncolytic virotherapy, Oncolytic viruses, Cancer immunotherapy, Viral engineering, Tumor microenvironment, Immunogenic cell death, Combination therapy, Precision oncology

## Abstract

Oncolytic virotherapy has emerged as a promising cancer treatment that combines selective tumor destruction with potent stimulation of antitumor immunity. Unlike conventional therapies, oncolytic viruses (OVs) preferentially infect and replicate within malignant cells while sparing normal tissues, leading to direct tumor lysis and the release of tumor-associated antigens, pathogen-associated molecular patterns, and damage-associated molecular patterns that activate both innate and adaptive immune responses. Advances in molecular virology and genetic engineering have transformed naturally occurring viruses into programmable therapeutic platforms capable of delivering immunomodulatory payloads that enhance antitumor efficacy and remodel the tumor microenvironment. Multiple DNA- and RNA-based viral platforms, including herpes simplex virus, adenovirus, vaccinia virus, reovirus, measles virus, Newcastle disease virus, vesicular stomatitis virus, and coxsackievirus, are currently under clinical investigation, with regulatory approval of talimogene laherparepvec (T-VEC) and G47Δ providing important proof of clinical feasibility. Increasing evidence indicates that OVs can convert immunologically "cold" tumors into "hot" tumors, thereby improving responsiveness to immune checkpoint inhibitors and other therapeutic modalities. Consequently, oncolytic virotherapy has become an attractive component of combination strategies involving immunotherapy, chemotherapy, radiotherapy, targeted therapies, and cellular therapies. Nevertheless, several challenges continue to limit its broader clinical application, including antiviral immune clearance, inefficient systemic delivery, restricted intratumoral spread, tumor heterogeneity, manufacturing complexity, and the lack of robust predictive biomarkers. This review provides a comprehensive overview of the biological foundations of oncolytic virotherapy, viral engineering strategies, mechanisms of tumor selectivity, direct oncolysis, immunogenic cell death, and immune remodeling of the tumor microenvironment. It further summarizes the major oncolytic virus platforms, recent preclinical and clinical advances, emerging combination strategies, current translational challenges, and future directions expected to advance precision oncolytic virotherapy and expand its role in modern cancer treatment.

**Graphical Abstract:**

**Figure:**
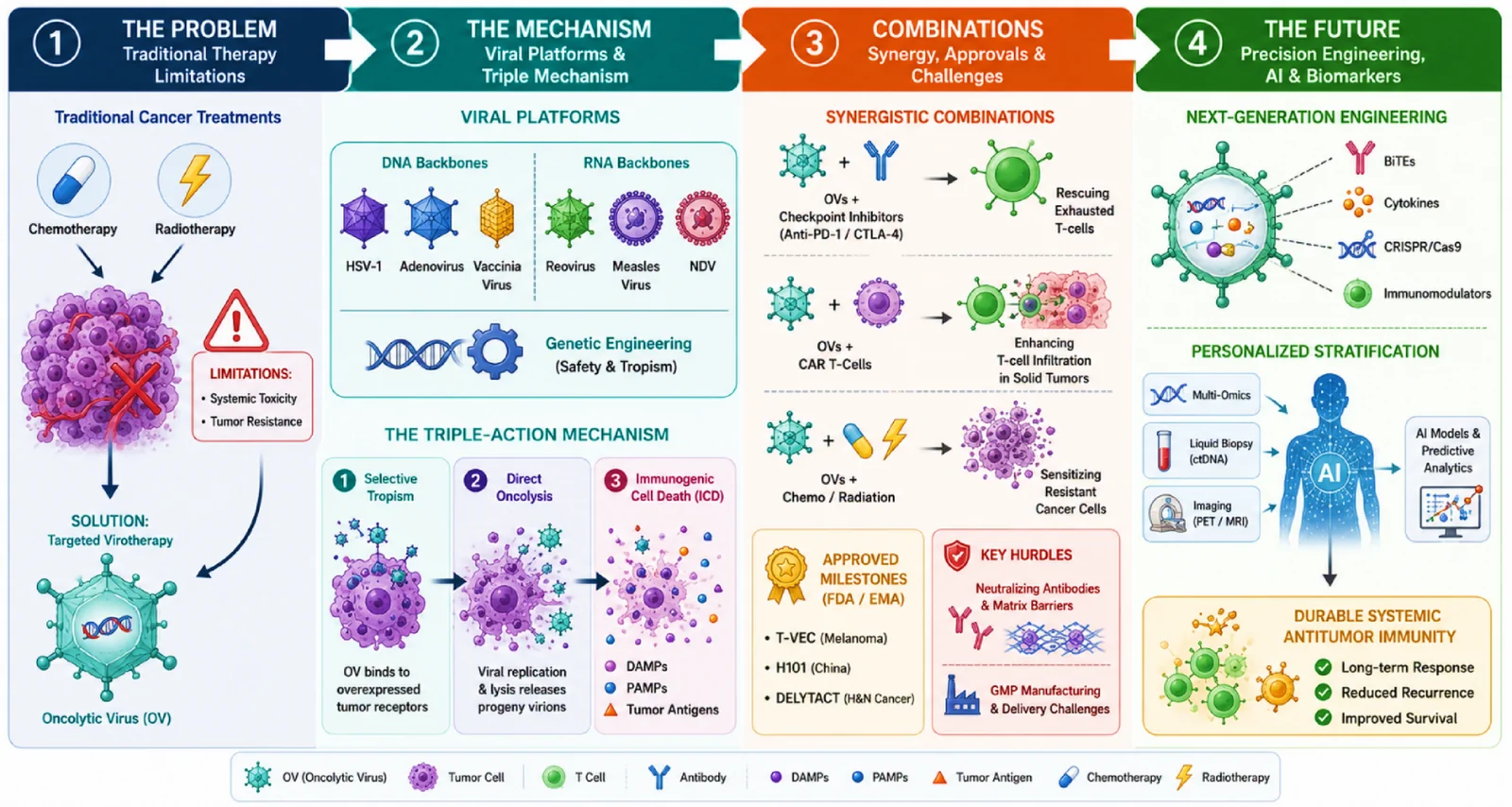

Comprehensive overview of oncolytic virotherapy (OVT) from fundamental biology to clinical translation

## Introduction

Cancer remains one of the leading causes of morbidity and mortality worldwide despite remarkable advances in diagnosis and treatment [1]. According to recent global estimates, nearly 20 million new cancer cases and approximately 10 million cancer-related deaths occur annually, and these numbers are expected to increase substantially over the coming decades owing to population aging and continued demographic growth [2]. Although improvements in screening, surgery, radiotherapy, systemic chemotherapy, targeted therapies, and immunotherapy have significantly improved patient outcomes in several malignancies, durable disease control remains elusive for many patients with advanced or metastatic cancer [3, 4]. Tumor heterogeneity, therapeutic resistance, immune evasion, and treatment-associated toxicities continue to limit the long-term effectiveness of current therapeutic strategies, highlighting the urgent need for more selective and durable anticancer approaches [5, 6].

Among recent advances in cancer therapy, immunotherapy has fundamentally changed the treatment landscape by harnessing the patient's immune system to recognize and eliminate malignant cells [7]. Immune checkpoint inhibitors, adoptive cell therapies, and cancer vaccines have produced remarkable and durable responses in selected patient populations. However, the clinical benefits of these approaches remain restricted to a subset of patients because many tumors possess an immunosuppressive microenvironment characterized by poor immune-cell infiltration, impaired antigen presentation, and multiple mechanisms of immune escape. These so-called "cold" tumors often respond poorly to existing immunotherapies, emphasizing the need for therapeutic strategies capable of simultaneously destroying tumor cells and stimulating effective antitumor immunity [8–10].

Oncolytic virotherapy has emerged as one of the most promising immunotherapeutic platforms to address these challenges [11]. Unlike conventional anticancer therapies that primarily target tumor cell proliferation or specific oncogenic pathways, oncolytic viruses (OVs) selectively infect, replicate within, and lyse malignant cells while largely sparing normal tissues. Viral replication within tumors not only induces direct cancer cell destruction but also initiates a cascade of immunological events that convert dying tumors into highly immunogenic sites. The release of tumor-associated antigens together with pathogen-associated molecular patterns (PAMPs) and damage-associated molecular patterns (DAMPs) promotes dendritic-cell activation, antigen presentation, and the subsequent induction of both innate and adaptive antitumor immune responses. Consequently, OVs function as dual-action therapeutics that combine direct oncolysis with immune-mediated tumor eradication [11, 12].

The concept of using viruses to treat cancer originated more than a century ago following observations that some patients experienced transient tumor regression after naturally acquired viral infections [13]. However, the clinical application of this concept remained limited until advances in molecular virology and genetic engineering enabled the development of viruses with enhanced tumor selectivity, improved safety, and greater therapeutic efficacy. These technological advances transformed naturally occurring viruses into programmable therapeutic platforms capable of delivering immunostimulatory molecules, cytokines, checkpoint inhibitors, tumor suppressors, and other therapeutic payloads directly to the tumor microenvironment [14–16]. Today, oncolytic virotherapy represents a rapidly evolving field at the intersection of virology, immunology, genetic engineering, and precision oncology [11].

A wide variety of DNA and RNA viruses have been developed as oncolytic platforms, including herpes simplex virus, adenovirus, vaccinia virus, reovirus, measles virus, Newcastle disease virus, vesicular stomatitis virus, and coxsackievirus. Each platform possesses distinct biological characteristics that influence tumor selectivity, genome engineering capacity, immune activation, and delivery strategies [17, 18, 19]. Among these, genetically modified herpes simplex virus has achieved the greatest clinical success, culminating in regulatory approval of talimogene laherparepvec (T-VEC) for advanced melanoma and G47Δ for malignant glioma in Japan. These milestones have provided important clinical proof of concept that engineered viruses can be translated into effective cancer therapeutics [20, 21].

Increasing evidence now indicates that the therapeutic activity of OVs extends far beyond direct tumor lysis. Viral infection remodels the tumor microenvironment by promoting immune-cell recruitment, enhancing antigen presentation, stimulating inflammatory cytokine production, and reversing local immunosuppression [11, 22, 23]. Through these mechanisms, OVs can convert immunologically "cold" tumors into "hot" tumors that are more responsive to immune checkpoint blockade and other immunotherapies. This ability has positioned oncolytic virotherapy as an attractive partner for combination treatment strategies involving immune checkpoint inhibitors, chemotherapy, radiotherapy, targeted therapies, cellular immunotherapies, and emerging precision medicine approaches [11, 16, 18].

Despite substantial progress, several important challenges continue to limit the widespread clinical implementation of oncolytic virotherapy. Host antiviral immunity can rapidly neutralize therapeutic viruses following systemic administration, reducing tumor delivery and persistence. In addition, the dense extracellular matrix, abnormal tumor vasculature, and highly heterogeneous tumor microenvironment can restrict viral dissemination within solid tumors [24, 25]. Manufacturing complexity, biomarker identification, optimal patient selection, and the design of effective combination regimens also remain major obstacles to broader clinical translation [26, 27]. Consequently, improving viral engineering, developing innovative delivery systems, identifying predictive biomarkers, and optimizing rational combination therapies have become major priorities in the next generation of OV research [27, 28].

Collectively, these advances have shifted the perception of oncolytic viruses from simple tumor-lytic agents to multifunctional immunotherapeutic platforms capable of integrating direct cytotoxicity with immune modulation. Rather than serving solely as stand-alone therapies, OVs are increasingly viewed as versatile components of multimodal cancer treatment designed to enhance the efficacy of existing therapeutic modalities while overcoming resistance mechanisms [11, 29].

In this review article, we provide a comprehensive overview of the biological principles underlying oncolytic virotherapy, including viral engineering strategies, mechanisms of tumor selectivity, direct oncolysis, immunogenic cell death, and immune remodeling of the tumor microenvironment. We further discuss the major oncolytic virus platforms, summarize current preclinical and clinical advances, evaluate combination strategies with established cancer therapies, examine the principal biological and translational challenges facing the field, and highlight emerging technologies that are expected to shape the next generation of precision oncolytic virotherapy.

## Principles and Mechanisms of Oncolytic Virotherapy

Oncolytic virotherapy is based on the ability of selected viruses to preferentially infect, replicate within, and destroy cancer cells while sparing most normal tissues. Unlike conventional anticancer therapies that primarily induce direct cytotoxicity, oncolytic viruses (OVs) exert their therapeutic effects through two complementary mechanisms. First, they selectively replicate in malignant cells, leading to direct tumor destruction. Second, viral infection transforms the tumor into an inflammatory microenvironment that stimulates innate and adaptive immune responses against the cancer. Consequently, the therapeutic efficacy of modern oncolytic virotherapy depends not only on efficient viral replication but also on the generation of durable systemic antitumor immunity [28, 30–34].

Current evidence supports a unified mechanistic model in which therapeutic activity proceeds through sequential but interconnected events. Initially, OVs selectively target susceptible tumor cells and establish productive viral replication. This replication results in direct tumor cell lysis and the release of newly generated viral particles, enabling infection to spread throughout the tumor. Simultaneously, dying cancer cells release tumor-associated antigens (TAAs), pathogen-associated molecular patterns (PAMPs), damage-associated molecular patterns (DAMPs), cytokines, and chemokines. Together, these molecules activate innate immune cells, promote antigen presentation, and stimulate tumor-specific T-cell responses that extend antitumor activity beyond the sites of viral infection. Thus, contemporary oncolytic virotherapy is increasingly recognized as both a targeted cytotoxic therapy and an in situ cancer vaccination strategy [30, 33, 35].

### Selective Tumor Targeting (Viral Tropism)

Selective tumor targeting represents the fundamental principle underlying oncolytic virotherapy. Successful treatment requires viruses to infect and replicate preferentially within malignant cells while minimizing infection of healthy tissues. This selectivity results from a combination of natural viral tropism, cancer-associated cellular abnormalities, characteristics of the tumor microenvironment, and genetic engineering approaches that further enhance tumor specificity [30, 34, 36, 37].

#### Natural viral tropism

Natural viral tropism refers to the inherent preference of a virus for specific cell types based on the interaction between viral surface proteins and host-cell receptors. Many cancers overexpress receptors that facilitate viral attachment and entry, making malignant cells more susceptible to infection than normal tissues [11, 38].

Several clinically important OVs exploit these receptor differences. Adenovirus serotype 5 primarily enters cells through the coxsackievirus and adenovirus receptor (CAR), whereas alternative adenoviral serotypes and chimeric vectors, including Ad3 and Ad5/35, preferentially utilize CD46 or desmoglein-2 (DSG2), thereby broadening their infectivity across different tumor types [16, 37, 39]. Measles virus targets CD46 and signaling lymphocytic activation molecule (SLAM), both of which are frequently upregulated in malignant tissues. Herpes simplex virus (HSV) enters cells through herpesvirus entry mediator (HVEM) and nectin-1 or nectin-2 receptors, whereas poliovirus selectively infects cells expressing CD155, a receptor that is highly expressed in numerous solid tumors and hematological malignancies [11, 18, 40]. Similarly, coxsackievirus A11 preferentially infects thoracic malignancies through intercellular adhesion molecule−1 (ICAM-1), illustrating how receptor expression can determine tumor susceptibility to specific viral platforms [41].

Although receptor abundance strongly influences viral entry, receptor expression alone does not determine therapeutic success. Productive infection also depends on the ability of tumor cells to support efficient viral replication after viral entry [11, 42].

#### Intracellular permissiveness

**Fig. 1:**
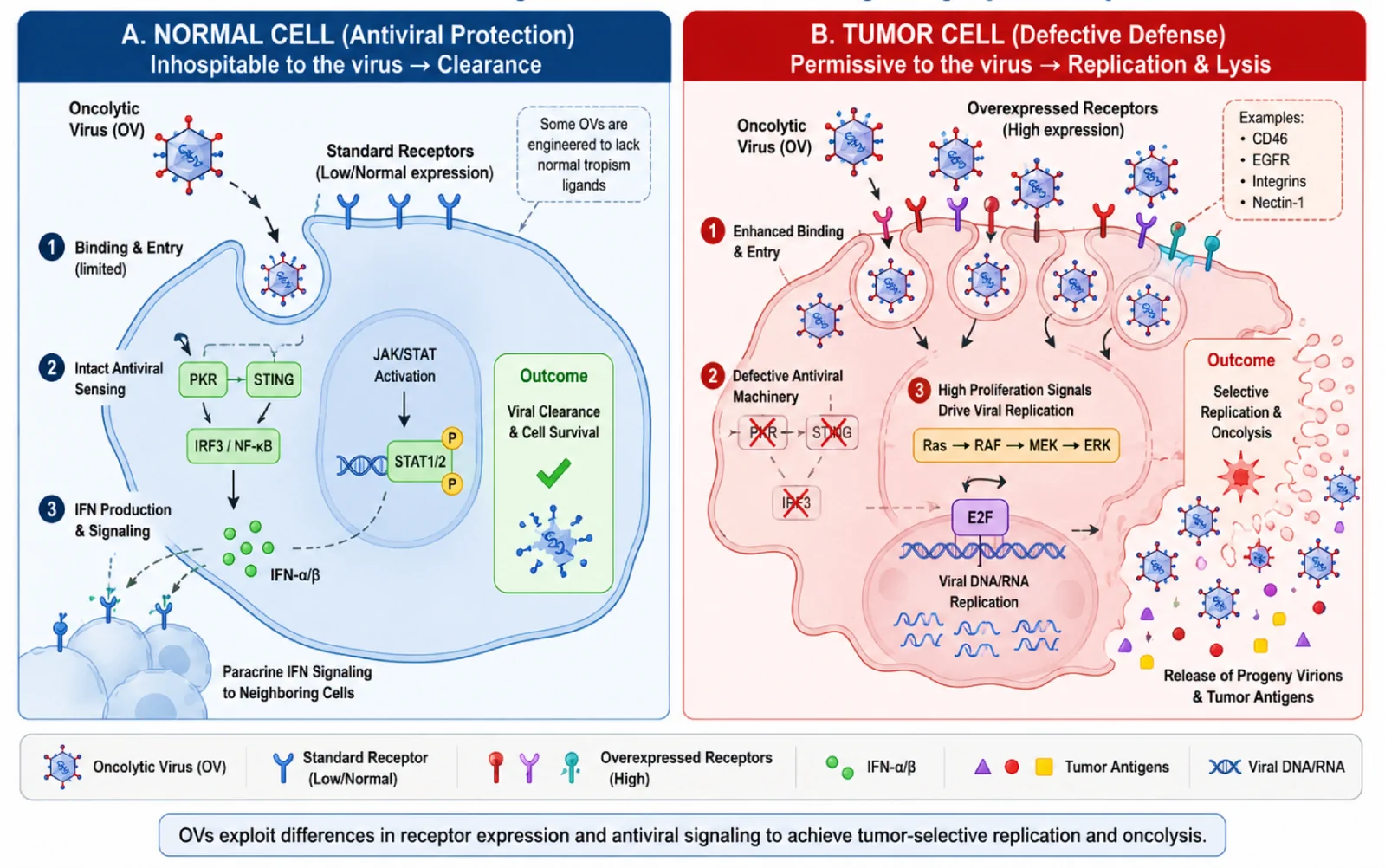
Mechanisms governing tumor-selective targeting by oncolytic viruses (OVs). *(Left)* In normal tissue, active antiviral defense pathways (such as intact IFN signaling and PKR activation) successfully restrict viral transcription, leading to rapid viral clearance and cell survival. *(Right)* In neoplastic cells, altered surface receptor profiles enhance viral tropism and entry. Concurrently, defective IFN/STING pathways and hyperactivated oncogenic signaling (e.g., Ras pathway) create a permissive intracellular environment, resulting in selective viral replication, mandatory host cell lysis, and progeny release

Following entry into the cell, OVs rely on the intracellular environment to complete their replication cycle (Fig. 1**)**. Cancer cells frequently harbor genetic and signaling abnormalities that unintentionally create conditions favorable for viral replication while weakening antiviral defense mechanisms [11, 43].

Activation of oncogenic pathways, including Ras, epidermal growth factor receptor (EGFR), Wnt/β-catenin, phosphoinositide 3-kinase (PI3K)/AKT, and defects in the p53, retinoblastoma (RB)/E2F, ATM, and apoptotic signaling pathways, supports viral replication by increasing cellular metabolic activity, promoting protein synthesis, and reducing antiviral responses [11, 36, 40]. Consequently, malignant cells become substantially more permissive to viral infection than normal cells.

Reovirus provides one of the best-characterized examples of this mechanism. In tumors with activated Ras signaling, antiviral protein kinase R (PKR) activity is suppressed, allowing efficient viral protein translation and replication. At the same time, Ras activation reduces type I interferon-mediated antiviral responses, enabling selective viral propagation within malignant cells while normal tissues retain effective antiviral protection [17, 18].

Similarly, many tumors exhibit defects in innate immune sensing pathways, particularly the cyclic GMP–AMP synthase–stimulator of interferon genes (cGAS–STING) pathway and type I interferon signaling. These defects impair the ability of cancer cells to detect and eliminate viral infection, thereby providing a permissive environment for OV replication. In contrast, healthy tissues generally preserve intact antiviral signaling, limiting viral replication and contributing to the favorable safety profile of many clinically developed OVs [30, 44, 45].

#### Influence of the tumor microenvironment

The tumor microenvironment (TME) further contributes to selective viral targeting. Unlike normal tissues, solid tumors are characterized by hypoxia, abnormal vascular architecture, nutrient deprivation, oxidative stress, and chronic inflammation. These conditions alter cellular metabolism and gene expression, creating an environment that often favors viral replication [33, 46].

Hypoxia is particularly important because activation of hypoxia-inducible factor-1 (HIF-1) signaling promotes transcriptional programs that enhance viral gene expression and replication in several OV platforms. In addition, impaired immune surveillance within many tumors reduces early viral clearance, allowing viruses to spread more efficiently through malignant tissue than through healthy organs [36, 45]. Consequently, both intrinsic cellular defects and unique microenvironmental conditions cooperate to enhance tumor-selective viral replication.

#### Engineering strategies to improve tumor selectivity

Although many OVs possess inherent tumor selectivity, modern virotherapy increasingly relies on genetic engineering to improve specificity, maximize therapeutic efficacy, and reduce off-target toxicity (Table 1). These modifications have transformed naturally occurring viruses into highly programmable therapeutic platforms [16, 17].

**Table 1:** Engineering strategies that enhance tumor-selective targeting of oncolytic viruses

Strategy	Mechanism	Representative examples	Principal advantage	References
Receptor retargeting	Redirects viral entry toward tumor-enriched receptors	Ad5/F35 vectors targeting CD46; EGFR-targeted adenovirus; EGFR-retargeted HSV-1 or modified HSV-1 gD variants optimizing nectin-1 entry	Improves tumor-specific viral entry	[49, 50]
Transcriptional targeting	Restricts viral gene expression using tumor-selective promoters	hTERT-driven adenoviruses; survivin promoter-controlled conditionally replicating adenoviruses	Enhances safety by limiting replication in normal tissues	[51, 52]
Replication detargeting	Viral gene deletions or tissue-specific microRNA regulation	First- and second-generation gene-deleted OVs; microRNA-regulated vectors	Reduces off-target viral replication	[53, 54]
Carrier-mediated delivery	Employs cellular carriers to enhance tumor localization	Mesenchymal stromal cell-mediated OV delivery	Improves biodistribution and protects viruses from immune clearance	[55–57]

Receptor retargeting modifies viral attachment proteins to redirect viral entry toward receptors that are preferentially expressed on tumor cells. Examples include adenoviral vectors engineered to utilize CD46 instead of CAR and HSV-1 vectors retargeted toward epidermal growth factor receptor (EGFR) or nectin-1, thereby improving infection of tumors with limited susceptibility to their native viruses [18, 40].

Transcriptional targeting restricts viral gene expression to malignant cells by placing essential viral genes under the control of tumor-selective promoters, such as those derived from human telomerase reverse transcriptase (hTERT) or survivin. Because these promoters exhibit minimal activity in most normal tissues, viral replication remains largely confined to cancer cells, thereby enhancing safety [47, 48].

Replication detargeting provides an additional level of control by deleting viral virulence genes or incorporating tissue-specific microRNA response elements that suppress viral replication in healthy tissues while preserving efficient replication within tumors. These approaches have substantially improved the therapeutic index of several first- and second-generation OV platforms [33, 47].

Targeted delivery strategies further enhance tumor specificity by improving viral biodistribution. Mesenchymal stromal cells (MSCs), for example, can serve as cellular carriers that protect OVs from immune neutralization during systemic administration and preferentially deliver them to tumor sites, thereby increasing intratumoral viral accumulation while reducing exposure of normal tissues [45].

Despite major advances in viral engineering, achieving uniform tumor targeting remains challenging. Tumors exhibit considerable interpatient, intrapatient, and intratumoral heterogeneity, resulting in variable expression of viral entry receptors across different lesions and even among neighboring cancer cells. Consequently, receptor-negative subpopulations may escape infection, limiting therapeutic efficacy despite optimized viral design [16, 40].

Another important limitation is that many receptors exploited by OVs, including EGFR, HER2, CD46, and CAR, are overexpressed rather than exclusively expressed in cancer cells. Their presence in normal tissues creates the potential for off-target infection and restricts the therapeutic window of receptor-targeted viruses [16]. Therefore, current research increasingly focuses on integrating multiple layers of targeting, including receptor specificity, transcriptional regulation, replication control, and targeted delivery, to maximize tumor selectivity while maintaining safety. These next-generation approaches are expected to improve the precision and clinical effectiveness of oncolytic virotherapy across diverse cancer types.

### Direct oncolysis

**Fig. 2:**
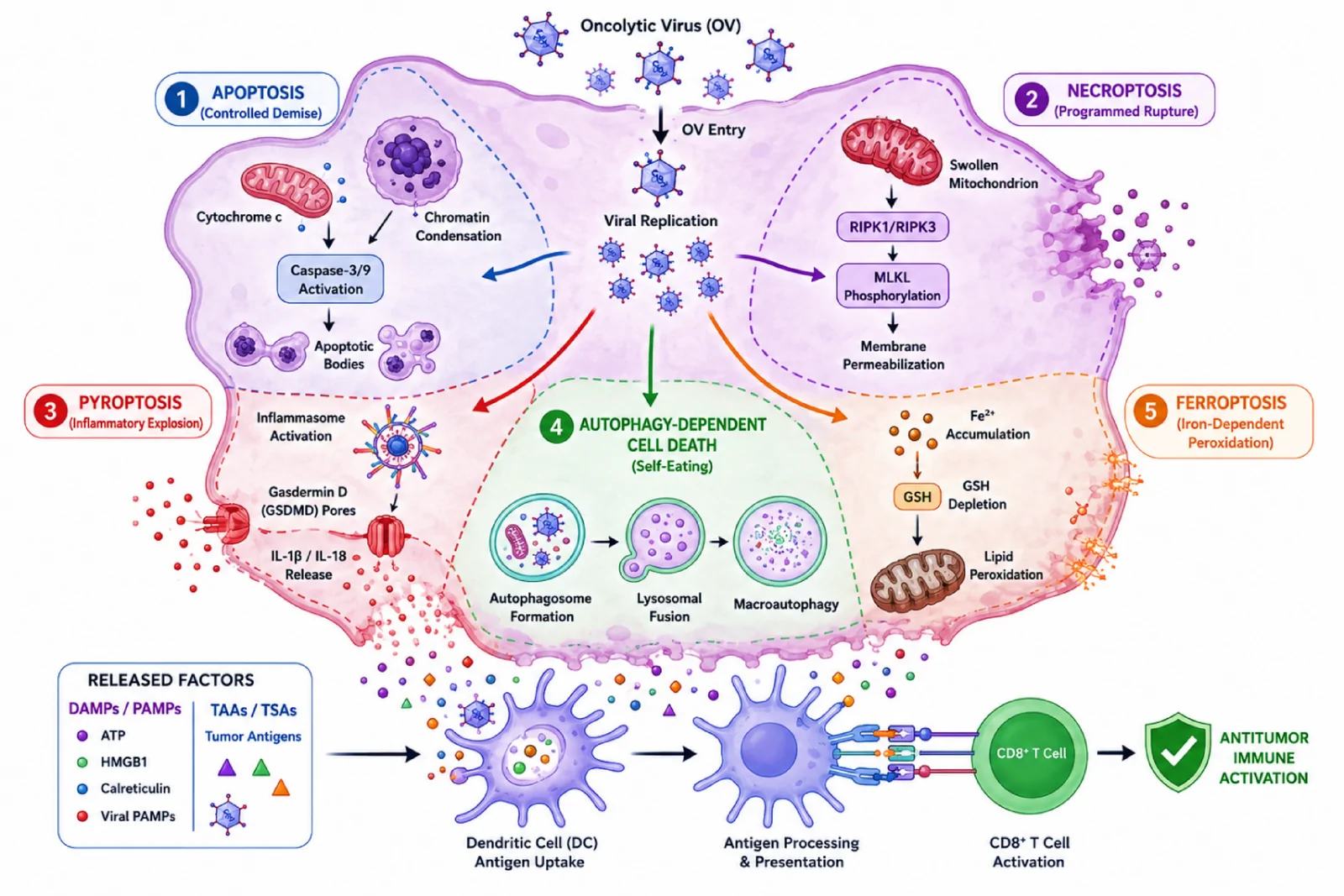
Major mechanisms of direct tumor cell death induced by oncolytic viruses. Oncolytic viruses eliminate cancer cells through multiple distinct, regulated cell death (RCD) pathways. Intracellular viral replication triggers classic apoptosis (mediated by caspases), necroptosis (driven by the RIPK1/RIPK3/MLKL axis), highly inflammatory pyroptosis (via gasdermin pore formation), autophagy-dependent cell death (characterized by autophagosome-lysosome fusion), and ferroptosis (driven by iron accumulation and lipid peroxidation). The synchronized execution of these pathways results in the massive release of tumor-associated antigens (TAAs), damage-associated molecular patterns (DAMPs), and pathogen-associated molecular patterns (PAMPs), fundamentally transforming the immunosuppressive tumor microenvironment and stimulating a robust systemic antitumor immune response

Direct oncolysis is the primary mechanism through which oncolytic viruses (OVs) eliminate malignant cells. Following successful entry into a susceptible tumor cell, the virus hijacks the host cellular machinery to replicate its genome, synthesize viral proteins, and assemble progeny virions. As viral replication progresses, infected cells lose membrane integrity and undergo lytic destruction, releasing newly formed virions that subsequently infect neighboring cancer cells (Fig. 2**)**. This continuous cycle of viral replication, tumor cell lysis, and local viral spread enables OVs to amplify their therapeutic effect within the tumor, distinguishing them from conventional therapies and non-replicating biological agents whose activity is limited to the administered dose [12, 22, 26, 33, 58].

Although direct tumor cell destruction is a defining feature of oncolytic virotherapy, the mechanisms underlying OV-induced cytotoxicity are highly diverse. Rather than activating a single mode of cell death, different viral platforms can trigger multiple regulated cell death pathways depending on the virus type, tumor genotype, and characteristics of the tumor microenvironment. Importantly, these pathways differ not only in their molecular mechanisms but also in their ability to stimulate antitumor immunity, making the quality of cell death as important as the extent of tumor lysis [31, 32].

Apoptosis is the most extensively characterized mechanism of OV-induced cell death (**(**Table 2**)**. Viral infection activates both the intrinsic (mitochondrial) and extrinsic (death receptor-mediated) apoptotic pathways, leading to sequential activation of caspases and controlled dismantling of the infected cell. Extrinsic apoptosis commonly involves activation of Fas, tumor necrosis factor receptor (TNF-R), or TNF-related apoptosis-inducing ligand receptor (TRAIL-R), followed by caspase−8 signaling, whereas intrinsic apoptosis results from mitochondrial dysfunction and cytochrome *c* release. Numerous OV platforms, including adenovirus, herpes simplex virus (HSV), Newcastle disease virus (NDV), and Semliki Forest virus (SFV), exploit these pathways to induce efficient tumor cell death. Furthermore, several engineered OVs have been designed to express pro-apoptotic molecules such as TRAIL or soluble Fas ligand (sFasL), thereby enhancing apoptotic signaling and increasing tumor-killing efficacy compared with their parental viral backbones [12, 17, 22, 59].

**Table 2:** Mechanisms of direct tumor cell death induced by oncolytic viruses

Cell death modality	Mechanistic features	Representative oncolytic viruses	Immunological consequence	References
Apoptosis	Caspase-dependent intrinsic and extrinsic programmed cell death	Adenovirus, HSV, NDV, SFV	Efficient tumor cell elimination with potential immunogenicity when accompanied by DAMP release	[40, 66]
Necroptosis	RIPK1/RIPK3/MLKL-mediated inflammatory programmed necrosis	Vaccinia virus, adenovirus, rVSV-NDV	Strong release of inflammatory mediators and enhanced immune cell recruitment	[60, 67, 68]
Pyroptosis	Inflammasome activation, gasdermin-mediated membrane pore formation, and cell lysis	Adenovirus, HSV-1	Potent activation of antigen-presenting cells and adaptive immunity	[69, 70]
Autophagy-dependent cell death	Excessive autophagic flux associated with viral stress responses	Adenovirus, NDV, hTERT-regulated CRAds	Promotes antigen processing and contributes to immunogenic cell death	[71–73]
Ferroptosis	Iron-dependent lipid peroxidation and reactive oxygen species accumulation	NDV (glioma models)	Emerging mechanism with distinct immunometabolic effects	[74, 75]

In addition to apoptosis, many OVs induce necroptosis, a regulated form of inflammatory cell death that serves as an important mechanism of tumor destruction. Unlike apoptosis, necroptosis culminates in plasma membrane rupture and the release of intracellular inflammatory mediators. This process is primarily regulated by receptor-interacting protein kinases RIPK1 and RIPK3 together with mixed lineage kinase domain-like protein (MLKL). Activation of these signaling molecules results in membrane permeabilization and the extracellular release of ATP, DNA, heat shock proteins (HSPs), and other immunostimulatory molecules that recruit and activate immune cells within the tumor microenvironment. Vaccinia virus (VV), adenovirus, and recombinant vesicular stomatitis virus expressing Newcastle disease virus glycoproteins (rVSV-NDV) have all been shown to induce necroptotic cell death. In particular, fusogenic viruses such as rVSV-NDV promote extensive syncytium formation that simultaneously activates apoptotic and necroptotic pathways, allowing effective viral replication and tumor destruction even when one death pathway is experimentally inhibited [12, 17, 22, 60].

Pyroptosis has recently emerged as another important mechanism contributing to OV-mediated tumor destruction. This highly inflammatory form of programmed cell death is characterized by inflammasome activation, cleavage of gasdermin proteins, formation of membrane pores, cellular swelling, and eventual membrane rupture. The resulting release of inflammatory cytokines and intracellular danger signals promotes maturation of antigen-presenting cells and enhances activation of tumor-specific adaptive immune responses. Increasing evidence indicates that several adenoviral and HSV-1 platforms can activate pyroptotic signaling, while c-Jun N-terminal kinase (JNK)-dependent pathways have also been implicated in OV-induced pyroptosis, highlighting its growing importance in cancer virotherapy [22, 61].

Autophagy-dependent cell death also contributes to the therapeutic activity of several OVs. Although autophagy is primarily a cellular survival mechanism that maintains metabolic homeostasis under stress, excessive or dysregulated autophagic activity during viral infection can result in irreversible cell death. In many OV systems, viral replication interacts closely with the host autophagic machinery to facilitate viral propagation while simultaneously promoting tumor cell destruction. Adenoviruses, NDV, and human telomerase reverse transcriptase (hTERT)-regulated conditionally replicating adenoviruses (CRAds) have all been reported to induce autophagy-associated tumor cell death. In addition to its direct cytotoxic effects, autophagy may enhance antigen processing and presentation, thereby strengthening subsequent immune responses and contributing to immunogenic cell death [48, 62].

More recently, ferroptosis has attracted considerable interest as an additional mechanism of OV-induced tumor killing. Ferroptosis is a distinct form of regulated cell death driven by iron-dependent lipid peroxidation and excessive accumulation of reactive oxygen species (ROS). Unlike apoptosis or necroptosis, ferroptosis is characterized by profound metabolic alterations and oxidative membrane damage. Although evidence remains limited, studies in glioma models have demonstrated that NDV infection can induce ferroptotic cell death, suggesting that this pathway may represent a promising therapeutic mechanism for future OV development, particularly in tumors resistant to conventional apoptotic signaling [59].

Importantly, these cell death pathways rarely occur in isolation. Instead, OV-induced oncolysis is typically a multimodal process in which several regulated cell death programs are activated simultaneously or sequentially within the same tumor. Adenoviral infection provides a representative example, as it can induce apoptosis, autophagy, necroptosis, and pyroptosis concurrently. Experimental studies further demonstrate that viral replication and tumor cell lysis often continue even when individual cell death pathways are pharmacologically or genetically inhibited, indicating substantial mechanistic redundancy. This flexibility allows OVs to maintain therapeutic activity despite the heterogeneous molecular alterations commonly observed among different tumors [40, 63, 64].

The ability to activate multiple forms of regulated cell death has important therapeutic implications. Although efficient viral replication remains essential for direct tumor destruction, increasing evidence indicates that the immunological characteristics of cell death are equally critical for long-term treatment success. Cell death pathways that promote the release of inflammatory mediators, tumor antigens, and danger signals generate stronger innate and adaptive immune responses than purely silent cytotoxicity. Consequently, the clinical efficacy of oncolytic virotherapy depends not only on the extent of direct tumor lysis but also on the capacity of virus-induced cell death to initiate durable systemic antitumor immunity. This concept provides the mechanistic foundation for the immunogenic cell death pathways discussed in the following section [11, 32, 41, 65]

### Anti-tumor immunity and Immunogenic Cell Death (ICD)

One of the most important advances in oncolytic virotherapy is the recognition that its therapeutic benefit extends far beyond direct tumor cell lysis. Although oncolytic viruses (OVs) eliminate cancer cells through productive viral replication, their greatest clinical value lies in their ability to initiate a potent antitumor immune response. Consequently, OVs are now regarded not only as tumor-selective cytolytic agents but also as in situ cancer vaccines that convert the patient's own tumor into a source of antigens and endogenous immune adjuvants capable of inducing systemic antitumor immunity [30, 41, 76].

**Fig. 3:**
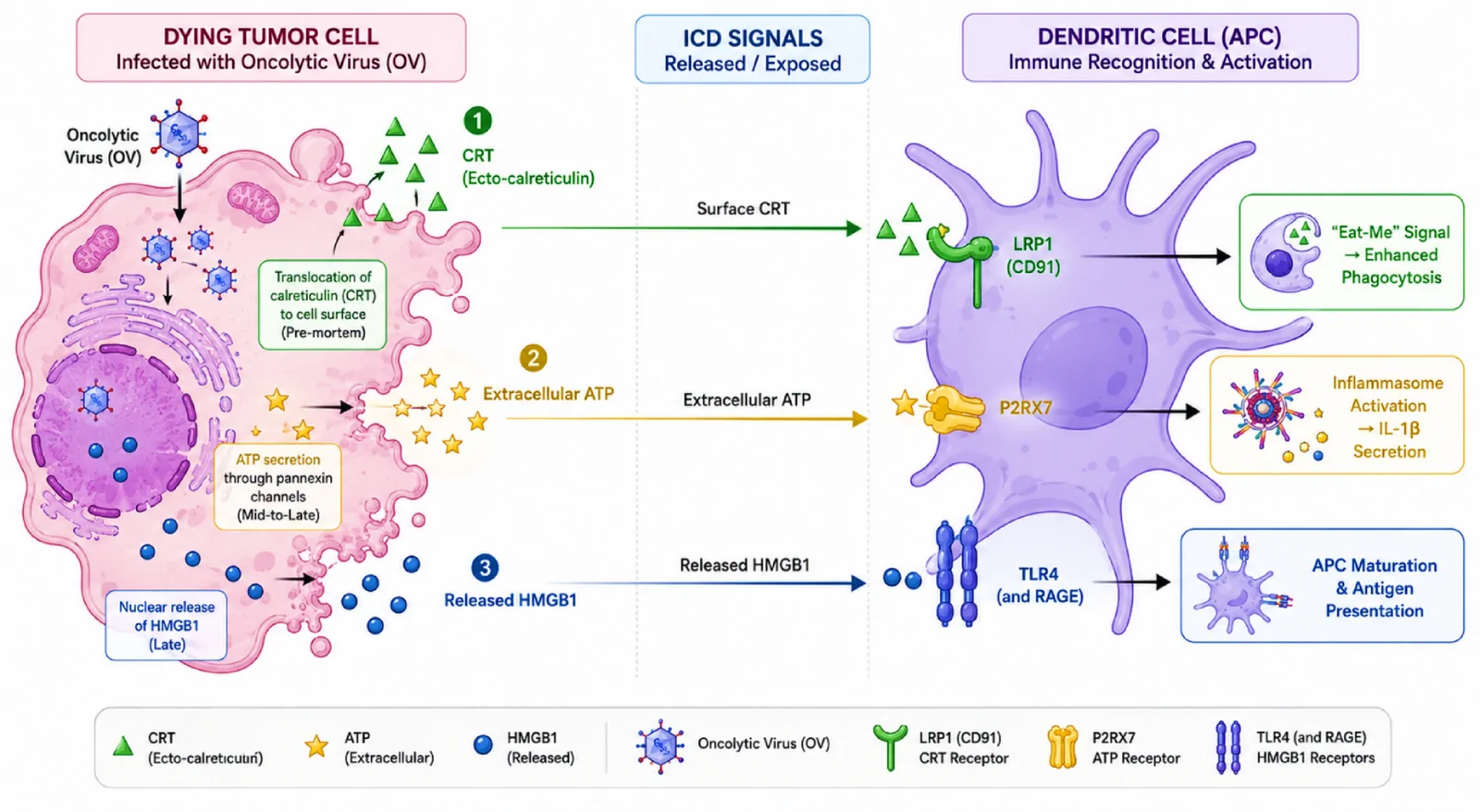
Canonical immunogenic cell death (ICD) signals induced by oncolytic viruses and their principal immune receptors. Oncolytic virus-mediated lysis drives the spatiotemporally coordinated release and exposure of key damage-associated molecular patterns (DAMPs) that serve as canonical ICD hallmarks. *(Top)* Surface-translocated calreticulin (ecto-CRT) acts as an crucial "eat-me" signal recognized by the LRP1 (CD91) receptor on dendritic cells, stimulating robust tumor cell phagocytosis. *(Middle)* Extracellularly secreted ATP binds to purinergic P2RX7 receptors, triggering inflammasome assembly and the subsequent release of pro-inflammatory IL-1beta. *(Bottom)* Passive release of the nuclear protein HMGB1 binds to toll-like receptor 4 (TLR4) and RAGE, promoting antigen-presenting cell (APC) maturation, cytokine production, and the initiation of a systemic, adaptive antitumor immune response

The transition from direct oncolysis to adaptive immune activation is primarily mediated through immunogenic cell death (ICD). Unlike non-immunogenic forms of cell death, ICD stimulates the immune system by coupling tumor cell destruction with the release of immunostimulatory signals (Fig. 3**)**. These signals recruit and activate antigen-presenting cells, promote tumor antigen processing, and initiate tumor-specific T-cell responses that can eliminate both infected and non-infected cancer cells. Thus, ICD represents the central link between viral replication and durable systemic antitumor immunity [62, 77].

#### Molecular hallmarks of immunogenic cell death

OV-induced ICD is characterized by the coordinated release or exposure of several highly conserved danger signals that alert the immune system to dying tumor cells. The four canonical hallmarks are surface exposure of calreticulin (CRT), extracellular release of adenosine triphosphate (ATP), release of high mobility group box 1 (HMGB1), and production of type I interferons (IFN-α and IFN-β). Together, these molecules promote efficient recognition of dying tumor cells, activation of innate immune responses, and initiation of adaptive immunity [41, 77, 78].

In addition to these canonical mediators, OV infection generates numerous damage-associated molecular patterns (DAMPs) and pathogen-associated molecular patterns (PAMPs). These include heat shock proteins (HSP70 and HSP90), viral double-stranded RNA (dsRNA), viral DNA and RNA, uric acid, extracellular histones, mitochondrial DNA, and circulating cell-free DNA (cfDNA). Because these molecules originate from both infected tumor cells and replicating viruses, they provide powerful activation signals for innate immune cells and amplify local inflammation within the tumor microenvironment [12, 23, 76].

#### Mechanisms of ICD signaling

The immunological effects of ICD are mediated through specific receptor–ligand interactions that coordinate the recruitment and activation of immune cells (**(**Table 3**)**. Surface-exposed calreticulin functions as an "eat-me" signal by binding to CD91 (also known as low-density lipoprotein receptor-related protein 1, LRP1) on dendritic cells and macrophages. This interaction promotes efficient phagocytosis of dying tumor cells and facilitates uptake of tumor-associated antigens for subsequent presentation to T lymphocytes [36, 78].

**Table 3:** Canonical immunogenic cell death signals induced by oncolytic viruses

ICD signal	Principal receptor	Major immunological function	References
Calreticulin (CRT)	CD91 (LRP1)	Promotes phagocytosis of dying tumor cells and antigen uptake	[79, 80]
ATP	P2Y2, P2X7	Recruits dendritic cells, activates the inflammasome, and promotes dendritic cell maturation	[41]
HMGB1	TLR4, RAGE	Enhances dendritic cell activation and antigen cross-presentation	[41, 81]
Type I interferons (IFN-α/β)	IFNAR and cGAS–STING-associated signaling	Activates NK cells, promotes T-cell priming, and amplifies antiviral and antitumor immunity	[82, 83]

Extracellular ATP serves as a potent chemoattractant that recruits dendritic cells to sites of viral infection through purinergic receptors, particularly P2Y2 and P2X7. ATP signaling also activates the NLRP3 inflammasome, promotes secretion of pro-inflammatory cytokines, and drives maturation of dendritic cells into highly effective antigen-presenting cells [36, 78].

HMGB1, a nuclear protein released during tumor cell lysis, binds pattern recognition receptors such as Toll-like receptor 4 (TLR4) and the receptor for advanced glycation end products (RAGE). Activation of these receptors enhances dendritic cell maturation, improves cross-presentation of tumor antigens, and supports the development of robust cytotoxic T-cell responses [22, 78].

Type I interferons constitute another essential component of ICD. Viral nucleic acids generated during OV replication are detected by intracellular pattern recognition receptors, including cyclic GMP–AMP synthase (cGAS), stimulator of interferon genes (STING), retinoic acid-inducible gene I (RIG-I), melanoma differentiation-associated protein 5 (MDA5), and Toll-like receptors (TLRs). Activation of these sensing pathways induces production of type I interferons, which stimulate natural killer (NK) cells, enhance dendritic cell function, promote antigen presentation, and support expansion and differentiation of tumor-specific CD8⁺ cytotoxic T lymphocytes [44, 78].

#### Activation of innate and adaptive antitumor immunity

The coordinated release of DAMPs, PAMPs, cytokines, and chemokines transforms the infected tumor into an inflammatory environment that supports immune cell recruitment and activation. Dendritic cells capture tumor-derived antigens released during viral oncolysis, undergo maturation, and migrate to draining lymph nodes where they present tumor peptides to naïve T cells through major histocompatibility complex (MHC) molecules. This process generates tumor-specific CD8⁺ cytotoxic T lymphocytes capable of recognizing and destroying malignant cells throughout the body, including tumor cells that have never been infected by the virus [11, 23, 76].

Simultaneously, NK cells recognize infected and stressed tumor cells through activating receptors and rapidly eliminate susceptible targets before adaptive immunity is fully established. Activated macrophages further amplify local inflammation by producing pro-inflammatory cytokines, chemokines, and additional danger signals that enhance recruitment of immune effector cells. Together, these innate and adaptive immune mechanisms establish a positive feedback loop that strengthens and sustains antitumor immunity [11, 66, 76].

One of the most important consequences of this immune activation is the conversion of immunologically "cold" tumors, which contain few infiltrating lymphocytes and respond poorly to immunotherapy, into "hot" tumors characterized by increased infiltration of CD8⁺ T cells, activated dendritic cells, NK cells, and inflammatory cytokines. This remodeling of the tumor microenvironment substantially improves responsiveness to immune checkpoint blockade and other immunotherapeutic strategies [22, 84, 85].

#### Evidence across different oncolytic virus platforms

The ability to induce ICD has been demonstrated across multiple OV platforms, although the magnitude and molecular characteristics of the response vary among viruses [11, 16]. HSV-1 RH2 induces classical ICD through surface exposure of calreticulin together with release of ATP and HMGB1, while activating both apoptotic and pyroptotic pathways in squamous cell carcinoma cells [61]. Talimogene laherparepvec (T-VEC), the first clinically approved OV, induces robust ICD characterized by calreticulin exposure, ATP secretion, HMGB1 release, activation of the cGAS–STING pathway, increased infiltration of CD8⁺ T cells, and regression of both injected and distant tumors in preclinical models [62, 86].

Similarly, the recombinant poliovirus PVSRIPO stimulates dendritic cells and macrophages through strong type I interferon signaling, resulting in efficient activation of tumor-specific adaptive immune responses [62]. Newcastle disease virus (NDV), vaccinia virus, adenovirus, and Semliki Forest virus (SFV) also induce ICD, although each virus activates distinct combinations of DAMPs, cytokines, and innate immune pathways. These differences highlight that the immunological properties of OVs are virus-specific and should be considered when selecting or engineering therapeutic platforms [62].

#### Systemic antitumor immunity and therapeutic implications

A defining feature of OV-induced immunity is its ability to generate systemic antitumor responses that extend beyond the site of viral administration. Following activation of tumor-specific T cells, immune effector cells circulate throughout the body and eliminate metastatic or non-injected lesions, a phenomenon commonly referred to as the abscopal effect. This systemic immune response is particularly important for patients with disseminated or metastatic disease, where local viral replication alone cannot eradicate all tumor deposits [12, 31, 86].

To further strengthen this immune response, many next-generation OVs have been engineered to express immunostimulatory transgenes. Examples include HSV vectors encoding granulocyte–macrophage colony-stimulating factor (GM-CSF), interleukin-12 (IL-12), interleukin-15 (IL-15), or single-chain antibodies targeting programmed cell death protein 1 (PD-1). These engineered viruses enhance dendritic cell activation, increase infiltration of cytotoxic T cells, promote long-term immunological memory, and improve control of distant tumors compared with their parental viral backbones [22, 31, 87].

Despite these advances, generating effective antitumor immunity requires careful balance between antiviral and antitumor responses. Innate immune sensing pathways, including cGAS–STING, RIG-I-like receptors, Toll-like receptors, and PKR, are essential for initiating ICD and promoting adaptive immunity. However, activation of the same antiviral pathways can also accelerate viral clearance, thereby limiting viral replication and reducing intratumoral spread [44, 88].

Additional challenges include heterogeneous expression of viral receptors, physical barriers created by the tumor stroma, differences in interferon responsiveness among tumors, and persistent immunosuppressive mechanisms within the tumor microenvironment. These factors contribute to variable therapeutic responses and help explain why OV monotherapy often produces incomplete clinical benefit [28, 58, 89].

Overall, the central mechanistic paradigm of oncolytic virotherapy has evolved from viewing OVs as selective tumor-lytic agents to recognizing them as powerful immune-modulating therapeutics. Through the induction of immunogenic cell death, OVs transform tumors into endogenous vaccines that activate durable systemic antitumor immunity. Future advances will depend on optimizing the balance between sustained tumor-selective viral replication and productive immune activation while minimizing premature antiviral clearance, thereby maximizing therapeutic efficacy across diverse and heterogeneous cancers.

## Key oncolytic virus platforms

The development of oncolytic virotherapy has been driven by a diverse collection of viral platforms that differ in genome organization, replication strategy, tissue tropism, immunogenicity, and engineering potential. Among these, herpes simplex virus (HSV), adenovirus, vaccinia virus, reovirus, measles virus (MV), and Newcastle disease virus (NDV) have emerged as the principal platforms for clinical and translational development [11, 16]. Although each virus possesses unique biological characteristics, a common principle has emerged across the field: native viral oncolysis alone rarely provides durable clinical benefit. Instead, therapeutic efficacy increasingly depends on genetic engineering strategies that improve tumor selectivity, enhance immune activation, overcome antiviral immunity, and enable rational combination therapies [16, 90].

To date, DNA viruses have dominated clinical translation because their relatively large and genetically stable genomes can accommodate substantial genetic modification while maintaining replication competence. Their large coding capacity enables the insertion of therapeutic transgenes, cytokines, immune checkpoint inhibitors, and other immunomodulatory payloads without compromising viral stability. Consequently, all currently approved oncolytic viruses, including the HSV-based talimogene laherparepvec (T-VEC) and G47Δ, as well as the adenovirus-based H101 (Oncorine), belong to the DNA virus family, highlighting the considerable translational advantages of these platforms [11, 16, 38].

### DNA viruses

#### Herpes simplex virus

Herpes simplex virus type 1 (HSV-1) is the most clinically advanced and extensively validated oncolytic virus platform. Its success largely reflects a favorable combination of biological and engineering characteristics, including a large double-stranded DNA genome, broad infectivity, efficient nuclear replication, and extensive non-essential genomic regions that permit the insertion of multiple therapeutic genes without increasing the risk of insertional mutagenesis [91–93]. These features have established HSV as the benchmark platform for the development of genetically engineered oncolytic viruses.

Modern HSV vectors are designed as conditionally replicating viruses that selectively replicate within malignant cells while sparing normal tissues. Tumor selectivity is typically achieved through deletion of viral neurovirulence genes, modification of viral replication pathways, and incorporation of therapeutic transgenes that amplify local immune responses. The best-known example is T-VEC, which contains deletions of ICP34.5 and ICP47 together with expression of granulocyte–macrophage colony-stimulating factor (GM-CSF). These modifications simultaneously enhance tumor specificity, improve antigen presentation, and stimulate local antitumor immunity, ultimately leading to its approval for advanced melanoma following successful phase III clinical trials [94, 95].

Subsequent generations of HSV vectors, including NV1020 [96], G207 [97], HSV1716 [98], and G47Δ [99], have incorporated increasingly sophisticated engineering strategies to optimize replication control, safety, and immunogenicity [93]. Collectively, these advances have transformed HSV from a naturally occurring neurotropic virus into a versatile immunotherapeutic platform capable of delivering multiple therapeutic payloads while simultaneously inducing direct tumor destruction and systemic immune activation.

#### Adenovirus

Adenoviruses represent one of the most extensively investigated oncolytic platforms owing to their efficient infection of proliferating cells, well-characterized genome, and exceptional flexibility for genetic manipulation [100, 101]. Unlike HSV, adenoviral vectors rely heavily on modifications that restrict viral replication to tumor cells through selective gene deletions, tumor-specific promoters, receptor retargeting, or capsid engineering. These approaches substantially improve tumor selectivity while minimizing toxicity to normal tissues [102].

Clinical development of adenoviral therapy has progressed from the approval of H101 (Oncorine) in China to several next-generation candidates, including CG0070 and OBP-301 (Telomelysin), many of which are currently being evaluated in combination with immune checkpoint inhibitors, chemotherapy, or radiotherapy [16, 103, 104]. Despite these advances, adenoviral efficacy remains limited by rapid immune clearance, inefficient intratumoral dissemination, and physical barriers within the tumor microenvironment. Consequently, current research increasingly focuses on improving systemic delivery, enhancing tumor targeting, and integrating adenoviruses into multimodal treatment strategies [16, 105].

#### Vaccinia virus

Vaccinia virus possesses several unique biological characteristics that distinguish it from other oncolytic platforms. It replicates exclusively in the cytoplasm, exhibits broad tumor tropism, accommodates exceptionally large therapeutic payloads, and demonstrates relatively efficient systemic dissemination following intravenous administration [106–108]. These properties make vaccinia particularly attractive for the treatment of disseminated metastatic disease.

Selective tumor replication is generally achieved through deletion of virulence genes, including thymidine kinase, thereby restricting efficient viral replication to metabolically active cancer cells while reducing pathogenicity in normal tissues [109]. Several engineered vaccinia viruses, including JX-594 (Pexa-Vec), Olvi-Vec, and TG6002, have demonstrated encouraging antitumor activity in preclinical and early clinical studies. Nevertheless, the disappointing phase III results of Pexa-Vec in hepatocellular carcinoma illustrate the complexity of translating promising biological activity into consistent clinical benefit [16, 110–112]. These findings emphasize that viral engineering alone is insufficient and that therapeutic success depends equally on patient selection, delivery strategies, and rational combination regimens [28, 29].

### RNA Viruses

#### Reovirus

Among RNA viruses, reovirus is the most clinically advanced naturally occurring oncolytic platform. Because reovirus preferentially replicates in transformed cells with dysregulated antiviral signaling pathways, particularly those involving activated RAS signaling, it can selectively destroy malignant cells while remaining largely non-pathogenic in healthy individuals [113, 114]. In addition to direct oncolysis, reovirus activates both innate and adaptive immune responses through induction of immunogenic cell death and inflammatory cytokine production [11, 115].

More than 50 clinical trials involving over 1,700 patients have evaluated reovirus across multiple cancer types, making it one of the most extensively studied RNA-based oncolytic viruses [113, 116]. However, as with other platforms, clinical efficacy as monotherapy has generally been modest. Current research therefore focuses on combination approaches with chemotherapy, radiotherapy, and immune checkpoint blockade, together with innovative delivery strategies such as nanoparticle encapsulation, oral formulations, and mesenchymal stem cell carriers to improve systemic administration and reduce immune-mediated viral clearance [117, 118].

#### Measles virus and Newcastle disease virus

Measles virus and Newcastle disease virus constitute the principal paramyxovirus platforms in oncolytic virotherapy [16, 119, 120]. Vaccine-derived measles virus strains have demonstrated encouraging safety profiles together with potent antitumor activity in both preclinical and clinical studies. Engineered variants, including MV-NIS and MV-CEA, further extend their clinical utility by incorporating reporter genes that enable non-invasive imaging while simultaneously enhancing therapeutic monitoring in malignancies such as multiple myeloma and ovarian cancer [16, 121–123].

Newcastle disease virus has attracted sustained interest because of its intrinsic tumor selectivity, strong immunogenicity, and ability to stimulate robust innate immune responses without causing severe disease in humans [124, 125]. Although both viruses demonstrate substantial biological promise, their broader clinical application remains constrained by limited systemic persistence and rapid neutralization by pre-existing antiviral immunity, particularly in the case of measles virus. To overcome these challenges, recent engineering strategies have focused on receptor retargeting, immune shielding, and improved delivery systems. For example, uPAR-retargeted measles virus has demonstrated enhanced tumor specificity with minimal off-target toxicity in preclinical models, illustrating the potential of precision viral engineering to expand the therapeutic window [11, 126–128].

#### Genetic engineering defines next-generation oncolytic viruses

Perhaps the most important advance in the field is the transition from naturally occurring or minimally attenuated viruses to highly engineered therapeutic platforms. Modern oncolytic viruses are no longer designed solely to replicate selectively within tumors but are increasingly programmed to remodel the tumor microenvironment, enhance immune activation, and synergize with other anticancer therapies [33, 129].

Across all major viral platforms, several engineering strategies have emerged as common design principles. These include deletion of virulence genes to improve safety, tumor-specific promoters to restrict viral replication, receptor and capsid retargeting to enhance tumor selectivity, microRNA-mediated detargeting to reduce off-target infection, and incorporation of immunostimulatory transgenes that amplify antitumor immunity [16, 26, 130].

Among these approaches, payload arming has produced some of the most significant therapeutic advances. Expression of GM-CSF by T-VEC provided the first clinical demonstration that local cytokine delivery could substantially enhance antitumor immunity [131, 132]. More recently, increasingly sophisticated constructs have combined multiple immune modulators within a single viral backbone. For example, vaccinia viruses expressing GM-CSF together with IL-21 or co-expressing IL-7 and IL-12 have demonstrated enhanced dendritic-cell activation, macrophage repolarization, increased T-cell infiltration, durable immune memory, and improved responsiveness to immune checkpoint blockade in preclinical models [133, 134]. Other engineered viruses have been designed to express synthetic receptors, tumor-associated antigens, bispecific T-cell engagers, or truncated CD19, thereby enabling synergistic interactions with CAR-T cell therapy and other forms of adoptive immunotherapy [135].

Improving systemic delivery has become another major engineering priority. Neutralizing antibodies rapidly eliminate many systemically administered viruses, particularly adenovirus, reovirus, and measles virus, thereby limiting repeated dosing and reducing therapeutic efficacy [136–138]. To overcome this obstacle, investigators are actively developing nanoparticle-based delivery systems, capsid shielding technologies, extracellular vesicle encapsulation, and cell-mediated delivery using mesenchymal stem cells and immune cells to protect therapeutic viruses during circulation and facilitate efficient tumor targeting [29, 139, 140].

Overall, the evolution of oncolytic virotherapy has shifted the focus from identifying naturally tumor-selective viruses to engineering multifunctional therapeutic platforms [11]. HSV currently represents the most clinically validated backbone, whereas adenovirus and vaccinia virus offer highly versatile DNA platforms with extensive engineering potential [141]. Reovirus, measles virus, and Newcastle disease virus continue to expand the RNA virus landscape and remain important candidates for next-generation virotherapy [18]. Future progress will depend less on the intrinsic biology of individual viruses than on continued advances in genetic engineering, precision targeting, immune modulation, and combination therapy that together maximize therapeutic efficacy while minimizing toxicity [11, 16].

## Clinical translation and combination strategies

### Approved OVs therapies

The field of oncolytic virotherapy has progressed substantially over the past two decades, transitioning from experimental preclinical models to multiple clinical trials, culminating in FDA approvals that mark a significant milestone in cancer immunotherapy. Despite decades of preclinical and clinical investigation, only a small number of OVs have achieved regulatory approval worldwide. This section reviews the currently approved oncolytic virotherapies, their mechanisms of action, pivotal clinical trial evidence, and their positioning within the broader landscape of cancer immunotherapy.

## Talimogene Laherparepvec (T-VEC)

Talimogene laherparepvec (T-VEC), marketed as IMLYGIC®, stands as the most widely approved and clinically utilized oncolytic agent worldwide. T-VEC is a genetically modified HSV-1 that has been engineered for enhanced tumor selectivity and immune stimulation [142]. It harbors the knockout of the neurovirulence gene ICP34.5 to attenuate replication in normal cells and enhance tumor selectivity, deletion of ICP47 to prevent viral suppression of antigen presentation and permit earlier expression of the US11 gene, and insertion of GM-CSF gene to promote the recruitment of APCs, thereby enhancing local and systemic antitumor immune responses [143, 144].

The development of T-VEC involved extensive preclinical characterization and clinical translation. The virus preferentially infects melanoma cells, replicating selectively within tumor tissue while exhibiting attenuated replication in normal cells. Upon viral replication, infected tumor cells undergo lysis, releasing both viral particles and tumor-associated antigens [145]. The presence of GM-CSF in this context appears to activate dendritic cells and promote a robust T-cell-mediated immune response. This therapeutic design represents a sophisticated approach to combining direct oncolysis with sustained immunological activation.

T-VEC preferentially replicates in tumor cells due to the engineered deletions of viral genes that normally suppress antiviral defenses in normal cells [146]. Upon viral replication, infected cells undergo multiple forms of cell death, including apoptosis, autophagy, and lytic necrosis, leading to release of viral progeny and tumor cell-derived antigens. T-VEC's armed transgene encoding GM-CSF plays a critical role in immune activation. GM-CSF, recruits and activates dendritic cells within the TME, which processes tumor-associated antigens released by virus-infected cells and present them to T cells, facilitating the priming of tumor-specific CD8 + T-cell responses. This local immune activation can translate into systemic antitumor immunity, as evidenced by the observation that T-VEC achieves responses in uninjected, distant lesions.

Clinical evidence supports the induction of systemic immunity by T-VEC. In neoadjuvant studies, T-VEC was shown to increase intratumoral CD8 + T-cell density threefold (p < 0.05) and upregulate programmed death-ligand 1 (PD-L1) expression [147]. These findings suggest that T-VEC reshapes the TME from an immune-excluded, "cold" tumor into an immune-infiltrated, "hot" tumor, potentially sensitizing previously resistant tumors to checkpoint inhibition. Similarly, single-cell analyses of T-VEC-treated patients demonstrated robust local and systemic immune responses, including tumor necrosis, activation of M1 macrophages, and infiltration of CD8 + effector memory T cells [148].

T-VEC was granted approval by the United States Food and Drug Administration (FDA) in October 2015 and subsequently by the European Medicines Agency (EMA) for the local treatment of unresectable cutaneous, subcutaneous, and nodal lesions in patients with melanoma recurrent after initial surgery [149]. The approval was supported by pivotal phase III OPTiM trial evidence showing clinical activity and durable responses, following promising phase I and II clinical trials [150–152]. The OPTiM clinical trial randomized 436 patients with unresectable stage IIIB–IV melanoma to intralesional T-VEC or subcutaneous GM-CSF [153]. The trial demonstrated a durable response rate (DRR) of 16.3% for T-VEC versus 2.1% for GM-CSF, with an overall response rate (ORR) of 26.4%, including a complete response (CR) rate of 10.8% and median overall survival (OS) 23.3 month *vs.* 18.9 month [154]. Notably, responses were observed in both injected and non-injected lesions, providing early evidence of systemic immunologic activity. This approval represented a landmark event, establishing T-VEC as the first-in-class oncolytic immunotherapy to enter routine oncology practice.

Real-world clinical experience has provided valuable context beyond controlled trials. A European multicenter analysis of 88 melanoma patients treated with T-VEC demonstrated an ORR of 63.7%, with 43.2% achieving C and 20.5% achieving partial response. Median treatment duration was 19 weeks with an average of 11 doses administered. Importantly, adverse events were predominantly mild, with grade 1 adverse events accounting for 64.1% of reported events. These data reinforce the favorable risk–benefit profile of T-VEC in the real-world setting [155].

A comprehensive analysis of 121 melanoma patients treated with T-VEC in routine clinical practice revealed an ORR of 67% (95% CI 58–76%), including CR in 35% of patients. Notably, this real-world cohort included heavily pretreated patients, with 87% having received at least one prior line of systemic therapy, indicating that T-VEC retains activity even in treatment-experienced populations. Median progression-free survival (PFS) was 12.2 months, and median OS was 35.5 months, with 95% of patients achieving CR maintaining disease-free status at median 19.1-month follow-up [156]. A comprehensive real-world analysis of 52 melanoma patients treated with T-VEC at a single institution found no cases of disseminated HSV infection [157]. Grade 3–4 cutaneous reactions were not documented, and treatment-related adverse events were predominantly mild.

The approved indication for T-VEC encompasses locally advanced and metastatic melanoma patients with unresectable disease at stage IIIB through IVM1a, specifically targeting cutaneous, subcutaneous, and nodal metastases amenable to intralesional injection [142]. While melanoma remains the primary indication, the success of T-VEC has prompted investigations into its application across multiple solid tumor types, including breast cancer, sarcoma, head and neck cancer, and other malignancies with injectable disease [139, 158].

### Additional Approved or Conditionally Approved Oncolytic Viruses

#### ECHO-7 (Rigvir®)

Rigvir is an immunomodulator derived from a non-pathogenic strain of the ECHO-7 enterovirus, was approved in Latvia in 2004 for the treatment of melanoma, skin and subcutaneous metastases of melanoma, and for prevention of relapse after radical surgery [159], making it historically the earliest regulatory approval among currently recognized oncolytic agents [160]. Its mechanism relies on natural tumor-selective tropism rather than genetic engineering.

Pre-registration efficacy studies involved 700 cancer patients [160]. Rigvir's approval and continued clinical use remain geographically restricted, and the agent has not undergone the large, randomized, controlled trials required for approval by major regulatory bodies such as the FDA or EMA. Post-marketing retrospective studies showed that Rigvir prolonged progression-free survival (PFS) by 6.67-fold compared to observation alone (P < 0.001) [160]. Another retrospective study reported a 4.39–6.57-fold lower mortality in Rigvir-treated stage IB–IIC melanoma patients compared with observation alone [159]. Additional case reports have described favorable outcomes in melanoma, small cell lung cancer, histiocytic sarcoma, and renal cell carcinoma [160].

#### Oncorine (H101)

Oncorine is a recombinant human adenovirus type 5, also known as H101, engineered with deletions in the E1B-55 kDa and partial E3 regions to enhance selective replication in p53-deficient tumor cells and to promote lysis-associated ICD. It was approved by the China Food and Drug Administration in 2005 for use in combination with chemotherapy for the treatment of refractory nasopharyngeal and head and neck carcinoma, representing the first government approval of an oncolytic adenovirus for cancer therapy [161]. Beyond its labeled indication, Oncorine has been investigated in exploratory studies across additional malignancies, including hepatocellular, pancreatic, cervical, and metastatic tumors, underscoring ongoing interest in broadening its clinical scope despite the restriction of formal approval to the Chinese market.

#### G47Δ (Teserpaturev/Delytact®)

Delytact (teserpaturev, G47Δ) is a third-generation, triple-mutated oncolytic HSV-1 originally engineered at Harvard Medical School and subsequently evaluated and approved for clinical use in Japan. It harbors three genetic modifications, deletions of ICP34.5, ICP6, and the α47 gene, to further enhance tumor selectivity, viral replication in cancer cells, and antigen presentation while preserving safety [162, 163]. Delytact received conditional and time-limited approval from Japan's Ministry of Health, Labour and Welfare in 2021 for the treatment of malignant glioma, based on a single-arm phase II trial demonstrating meaningful improvement in one-year survival among patients with residual or recurrent glioblastoma [164]. G47∆ was designated as a “Sakigake” breakthrough therapy in Japan, meaning “ahead of the world” [165]. This approval was notable both as the first oncolytic virus for a central nervous system malignancy and as an example of a conditional, real-world-data-contingent regulatory pathway increasingly relevant to advanced therapeutics with small trial populations [166].

### OVT in combination therapy

#### Combination with Immune checkpoint inhibitors

The convergence of OVT and immune checkpoint inhibition (ICI) represents one of the most clinically mature combination strategies, with compelling preclinical rationale supported by emerging clinical evidence. ICI monotherapy is most effective in tumors with pre-existing T-cell infiltration; in immunologically cold tumors, ORRs to PD-1/PD-L1 blockade alone are reported as low as 5–8%, compared with 30–40% in T-cell-inflamed tumors [167]. OVs induce ICD and innate immune activation sufficient to drive lymphocytic recruitment into the tumor, while ICIs reverse the T-cell exhaustion and compensatory PD-1/PD-L1 upregulation that oncolysis itself frequently provokes [167]. This orchestration of innate and adaptive immunity overcomes the limitations inherent to either approach used alone.

Clinical proof of concept was established in the phase Ib/III trial of T-VEC combined with pembrolizumab in advanced melanoma: the phase Ib cohort (n = 21) achieved an ORR of 61.9% and a CR of 33.3%, with grade 3/4 treatment-related adverse events occurring in approximately one-quarter of patients and no unanticipated safety signals. Notably, responses were observed in visceral metastases that had not been directly injected, providing the evidence that the combination confers systemic, rather than purely locoregional antitumor activity [167].

A pivotal randomized phase II study (n = 198) evaluated T-VEC plus ipilimumab (anti-CTLA-4) versus ipilimumab monotherapy in patients with advanced melanoma [168]. The combination regimen demonstrated a significantly improved ORR (35.7% vs. 16.0%) and DRR (33.7% vs. 13.0%) compared to ipilimumab alone. Median duration of response among objective responders was 69.2 months with the combination, representing an exceptionally durable response. The estimated 5-year OS was 54.7% in the T-VEC plus ipilimumab arm versus 48.4% in the ipilimumab-alone arm. Importantly, no new safety signals were observed, indicating that the combination was well-tolerated.

A phase III randomized trial (n = 692) compared T-VEC plus pembrolizumab (anti-PD-1) versus placebo plus pembrolizumab in treatment-naïve patients with advanced melanoma [169]. Combination with of T-VEC did not significantly improve the primary endpoints of PFS (HR 0.86; p = 0.13) or OS (HR 0.96; p = 0.74) compared to pembrolizumab monotherapy. However, ORR favored the combination (48.6% vs. 41.3%), and DRR was higher in the T-VEC group (42.2% vs. 34.1%). This unexpected finding raises important questions about patient selection and the timing of combination strategies in the PD-1 inhibitor-naive versus -exposed populations.

In contrast, a phase II study (SWOG S1607) evaluated T-VEC plus pembrolizumab specifically in melanoma patients who had progressed on prior PD-1/L1 inhibitor therapy [170]. Among patients with non-visceral metastases (n = 27), T-VEC plus pembrolizumab achieved a confirmed ORR of 26%, compared to 0% in the visceral disease cohort (n = 11). This suggests that T-VEC may be particularly effective in patients with limited disease burden and previous checkpoint inhibitor failure, potentially through mechanisms of immune reactivation and microenvironmental remodeling.

A neoadjuvant trial (NIVEC, n = 24) investigated T-VEC plus nivolumab in patients with resectable stage IIIB-IV melanoma with injectable disease prior to surgical resection [171]. The overall pathological response rate was 74%, with major pathological response achieved in 65% of patients. One-year event-free survival was 75%, suggesting that neoadjuvant T-VEC plus checkpoint inhibition may improve recurrence-free survival in resectable disease.

Comparable synergistic efficacy has subsequently been reported across melanoma, head and neck squamous cell carcinoma, breast cancer, hepatocellular carcinoma, and triple-negative breast cancer [172]. For example, phase I trial data from combination neoadjuvant T-VEC with systemic nivolumab (anti-PD-1) and ipilimumab (anti-CTLA-4) in HER2-negative breast cancer demonstrated pathologic CR in 17% of treated patients despite the triple immunotherapy regimen's associated toxicities, including grade 3 hypotension and autoimmune complications in some patients [173]. These results emphasize both the immunological power and the safety considerations of multi-modal combination approaches.

In high-grade glioma, where checkpoint blockade monotherapy performs poorly against an intrinsically immunosuppressive TME, preclinical models combining OVs with checkpoint inhibitors have demonstrated consistent increases in tumor-infiltrating lymphocyte density, although clinical validation in this indication remains at an earlier stage of development [174]. It should be emphasized, however, indications other than melanoma currently rely on smaller-cohort trials or preclinical models rather than confirmatory phase III evidence. Limited data exist for T-VEC in other malignancies. A phase II study of T-VEC and pembrolizumab in advanced sarcoma (n = 21 in expansion cohorts) reported best ORR of 11% for undifferentiated pleomorphic sarcoma/myxofibrosarcoma, 43% for cutaneous angiosarcoma, and 0% for epithelioid sarcoma. The cutaneous angiosarcoma cohort demonstrated particularly DR, with 71% achieving best ORR and 2 patients maintaining responses for 1–2 years or longer[175].

Another example is combining modified vesicular stomatitis virus (VSV) with PD-1 blockade in head and neck squamous cell carcinoma. VSV-S, engineered to express the pro-apoptotic SMAC/DIABLO gene, not only increases direct oncolysis but also triggers PANoptosis—a phenomenon encompassing pyroptosis, apoptosis, and necroptosis occurring through caspase−1/gasdermin D signaling [176]. This multi-death pathway induces robust CD8 + T-cell infiltration and enhances their cytotoxic capacity, and when combined with PD-1 blockade, produces significantly more potent anti-tumor effects than either therapy alone.

#### Combination with cytotoxic chemotherapy and radiotherapy

Conventional cytotoxic modalities were historically considered incompatible with OVT, based on the assumption that chemotherapy-induced immunosuppression would attenuate viral replication or inhibit antiviral immune priming. However, rather than uniformly suppressing the antitumor immune response elicited by oncolysis, selected chemotherapeutic regimens appear to sensitize tumors to subsequent checkpoint blockade when administered in conjunction with virotherapy. In a colorectal adenocarcinoma model, combined chemotherapy and oncolytic virotherapy sensitized tumors to checkpoint inhibition via a mechanism dependent on conventional type 1 dendritic cells [177].

A phase Ib study in advanced HER2-negative breast cancer (n = 19) evaluated T-VEC combined with chemotherapy or endocrine therapy in patients with injectable locoregional or chest wall disease [178]. Among 16 evaluable patients, partial responses were observed in 2 (13%), stable disease in 5 (31%), and progressive disease in 9 (56%). Notably, 61% of patients demonstrated response at the injected lesion sites by clinical assessment. Grade 3–4 treatment-related adverse events were generally manageable, with neutropenia being the most common, occurring in 26% of patients.

Radiotherapy is combined with OVs on a related but mechanistically distinct basis, where ionizing radiation induces ICD and can upregulate viral entry-receptor expression on tumor cells, while the virus supplies innate immune activation and antigen release that radiotherapy alone frequently fails to generate at a therapeutically meaningful magnitude [117, 174]. A phase IB/II trial (n = 30) combined intratumoral T-VEC with preoperative external beam radiation therapy in patients with locally advanced soft-tissue sarcoma of the extremities and trunk. No dose-limiting toxicities were observed, and adverse events were consistent with previously reported T-VEC safety profiles. Notably, 24% of evaluable patients achieved 95% pathological necrosis, and 2-year PFS and OS were 57% and 88%, respectively. No patients developed local recurrence after surgical resection, suggesting potential synergy between virotherapy and radiation [179].

#### Combination with adoptive cellular therapies: CAR-T and related platforms

The combination of oncolytic virotherapy with engineered cellular therapies—particularly chimeric antigen receptor (CAR) T cells and natural killer (NK) cells—represents an emerging frontier with profound implications for solid tumor treatment. Though CAR-T cell therapy has transformed outcomes in hematological malignancies but faces substantial barriers in solid tumors, including poor lymphocyte infiltration, immunosuppressive microenvironments, antigen heterogeneity permitting immune escape, and an immunosuppressive TME [78, 180]. OVs have been engineered to address multiple simultaneous barriers through their capacity to lyse heterogeneous tumor cells, release broad antigen repertoires, and remodel immunosuppressive stroma [180].

One strategy involves forced ectopic expression of a surrogate antigen, most commonly CD19, on the surface of infected solid-tumor cells, which has shown efficacy in preclinical melanoma and colon carcinoma models [181]. A second, more clinically advanced strategy arms the virus with immunomodulatory transgenes — chemokines such as CCL5, cytokines including IL-12 or IL-15, or anti-PD-1 antibody fragments — engineered to enhance CAR-T trafficking, intertumoral persistence, and effector function rather than to generate a de novo antigenic target [153, 156].

The oncolytic HSV-1 construct T7011, engineered to co-express CD19, CCL5, IL-12, and an anti-PD-1 payload, has demonstrated synergistic antitumor activity in combination with both CD19- and BCMA-directed CAR-T cells across multiple solid-tumor models and has since advanced into early-phase clinical evaluation [181, 182]. This approach transforms immunologically "cold" solid tumors into artificial "hot" tumors displaying universally recognized target antigens. In glioblastoma, oncolytic HSV-1 has been shown to potentiate the activity of CD70-directed CAR-T cells by augmenting intratumoral T-cell infiltration and interferon-γ release, establishing a rational combinatorial strategy for a tumor type in which CAR-T monotherapy has thus far demonstrated limited efficacy [180].

These studies reflect progressive shift toward engineering combinatorial activity directly into the viral construct rather than co-administering independently dosed agents. Transgene-armed vectors expressing GM-CSF (as exemplified by T-VEC), IL-12, chemokines, bispecific T-cell engagers, or checkpoint-blocking antibody fragments, exert their function as pre-integrated combination therapeutics, circumventing several of the pharmacokinetic mismatches and additive-toxicity complications associated with independently scheduled multi-agent regimens. This design philosophy reflects a broader conceptual repositioning of OV from a stand-alone cytotoxic modality in competition with chemotherapy or targeted agents, to a delivery and immune-priming platform explicitly engineered for combinatorial application from the outset [11, 117].

The clinical development landscape of oncolytic virotherapy, as outlined in ClinicalTrials.gov, identified 171 trials worldwide testing oncolytic virus-based therapies. The current pipeline draws on a broad range of viral platforms, adenoviruses, HSV-1, vaccinia virus, reovirus, measles virus, poliovirus, coxsackievirus, parvovirus, and vesicular stomatitis virus, reflecting sustained interest in exploiting the distinct biological properties and mechanisms each virus offers. It is reported that 62 clinical trials have been completed, while the remaining 109 are still underway or in other active phases [183].

## Challenges and hurdles in oncolytic virotherapy

Despite these advantages, several challenges have limited the widespread adoption of OV. These include incomplete tumor penetration, pre-existing host immunity against certain viral vectors, immune evasion mechanisms employed by tumor cells, and the immunosuppressive TME. Nevertheless, recent clinical successes and an expanding pipeline of engineered viruses suggest that optimized delivery strategies, rational combination approaches, and patient selection based on biomarkers may overcome these obstacles.

### Delivery and systemic spread

The most common clinical approach of currently approved oncolytic agents is direct intratumoral injection, which is a delivery constraint limiting therapeutic applicability to superficial, accessible lesions and is inadequate for treating metastatic disease [183]. Systemic intravenous administration despite showing remarkable oncolytic efficacy in in vitro and xenograft models, encounters passive sequestration and active immune-mediated mechanisms including near-immediate neutralization by pre-existing antibodies, complement-mediated inactivation, and rapid phagocytic clearance by macrophages and Kupffer cells, collectively eliminating a substantial proportion of the administered dose prior to tumor localization [56]. In addition, the TME poses further constraints of aberrant, poorly perfused vasculature, dense stroma, and elevated interstitial fluid pressure that collectively restrict both viral extravasation and subsequent intratumoral dissemination [29].

Several vector-engineering strategies are under active investigation as means of circumventing, these constraints. Cell-based carriers — mesenchymal stem cells, T lymphocytes, neural stem cells, and other populations exhibiting intrinsic tumor tropism — can shield viral particles from circulating antibodies while conferring a degree of active tumor-homing capacity, functioning as a biological delivery vehicle. However, substantial heterogeneity across cell types and experimental conditions in the existing literature precludes firm conclusions regarding the optimal carrier system for a given tumor context [57]. Chemical and polymer-based masking strategies, including glycosylated polyethylene glycol (PEG) coatings, aim to achieve comparable immune shielding without the added biological complexity of a cellular vehicle, with preclinical models reporting improved tumor targeting and enhanced immune cell infiltration [184]. Other approaches include nanoparticle co-loading, which combines virus with immunomodulatory agents or cytotoxic chemotherapeutics within a single delivery vehicle, and genetically engineered viral retargeting, which modifies capsid or envelope proteins to redirect tropism away from off-target organs toward tumor-specific receptors [136, 183]. None of these strategies has yet achieved sufficient validation at scale to displace intratumoral injection as the prevailing clinical standard,

### Tumor resistance mechanisms

The TME presents a fundamental biological paradox: the antiviral immune responses that OVs trigger to eliminate tumor cells simultaneously restrict viral replication and spread. Tumor cells can acquire heritable resistance to oncolytic infection, most commonly through chronic activation of the type I interferon signaling axis. In a preclinical model, oncolytic-virus-resistant melanoma clones derived following repeated viral exposure exhibited persistent upregulation of interferon-stimulated genes even in the absence of ongoing infection, which conferred cross-resistance to unrelated viral families, suggesting that the underlying resistance mechanism constitutes a generalized antiviral state rather than a virus-specific adaptation [185].

A related and more recently characterized mechanism implicates interferon regulatory factor 7 (IRF7) as a master regulator of this resistant phenotype in a murine colon carcinoma model. IRF7 upregulation both restricted viral replication directly and suppressed antitumor immunity via concomitant PD-L1 upregulation and CD80/CD86 downregulation on tumor cells, thereby achieving immune evasion through two convergent mechanisms [34, 186]. The cyclic GMP-AMP synthase–stimulator of interferon genes (cGAS-STING) cytosolic DNA-sensing axis can additionally restrict oncolytic HSV-1 replication, yet this same pathway appears necessary to sustain the adaptive antitumor immunity underlying the efficacy of combination checkpoint-inhibitor regimens [187]. This paradox between suppressing antiviral defense mechanisms to favor viral replication and preserving the immunogenic consequences of that same signaling represents one of the more conceptually challenges confronting the field, as it suggests that resistance and immunogenicity may not constitute independently addressable engineering targets.

Additionally, intratumoral heterogeneity poses a fundamental challenge, where highly diverse resistance mechanisms are deployed by different clonal populations within tumors. While one resistant clone might depend primarily on interferon-mediated responses, another might achieve resistance through cytoskeletal reorganization, altered expression of entry receptors, or small GTPase signaling changes [188]. Resistance is not confined to interferon signaling in isolation. Investigations of oncolytic parvovirus in pancreatic cancer models have demonstrated that certain tumor cells undergo a broader, late-phase collapse of innate immune homeostasis, characterized by concomitant suppression of interferon-stimulated genes and endogenous retroviral elements rendering affected cells refractory even to exogenous interferon rescue [189]. Collectively, this body of evidence indicates that tumor-intrinsic resistance to OVT is neither infrequent nor mechanistically uniform, as it it may be constitutive or acquired, pathway-restricted or genome-wide in scope.

### Manufacturing and regulatory considerations

The manufacture of OVs represents a fundamentally complex biopharmaceutical challenge characterized by competing demands for high yield, product consistency, regulatory compliance, and cost-effectiveness. Unlike recombinant protein therapeutics produced through standardized fermentation, OV manufacturing inherently depends on eukaryotic cell culture systems, which introduce substantial biological variability in addition to downstream purification procedures [190]. Viral products require phase-appropriate Good Manufacturing Practice (GMP) development pathways that differ substantially from conventional biologics as transitioning from research-grade to GMP-compliant manufacturing involves more than process optimization; it requires comprehensive documentation establishing that the manufacturing process consistently produces products meeting all pre-specified quality attributes [191]. A recurring and somewhat paradoxical technical obstacle is that the same neutralizing antibodies constraining systemic delivery in patients also interfere with conventional biosafety and detection assays during manufacturing, complicating quality-control workflows and motivating a shift toward next-generation-sequencing-based safety testing as a partial mitigation strategy.

The cumulative manufacturing complexity translates into formidable cost barriers including cost infrastructure supporting GMP manufacturing, quality control and regulatory compliance. Scaling to serve global patient populations demands manufacturing platforms substantially more efficient and cost-effective than current systems including revolutionary improvements in cell culture productivity and development of fundamentally novel manufacturing platforms [26].

Regulatory approval of oncolytic viruses as therapeutic agents encounters unique challenges stemming from their status as "living therapeutics"—biological entities capable of replication, mutation, and complex interactions with host systems which requires comprehensive OV risk assessment more complicated than traditional regulatory frameworks developed for chemotherapy agents, monoclonal antibodies, or recombinant proteins [191].

### Additional considerations: safety, biomarkers, and access

Ensuring safety in normal tissue frequently occurs at the direct expense of replicative and oncolytic potency, an inherent trade-off that every engineered platform must navigate rather than resolve definitively [56]. There is an urgent need for validated, prospectively qualified predictive biomarkers enabling patient selection; in the absence of such biomarkers, negative trial outcomes are correspondingly difficult to interpret, since non-responders cannot yet be reliably distinguished from biologically resistant tumors. Integration of artificial intelligence and machine learning approaches with comprehensive genomic, proteomic, and immune profiling may eventually enable such biomarker discovery, yet these approaches lack clinical standardization [192]. Geographic and economic access considerations remain similarly unresolved: clinical trials and product access remains concentrated within a limited number of economically developed health systems, and the substantial per-batch manufacturing costs are likely to reinforce this disparity [193].

Delivery, resistance, manufacturing, and regulatory strategy are increasingly interdependent: a masking strategy that improves systemic delivery may compromise a biosafety assay; a vector engineered to suppress antiviral resistance may concomitantly attenuate the immune priming upon which checkpoint-inhibitor combination efficacy depends; and a manufacturing process validated for an early-phase cohort may not scale cleanly to the randomized, adequately powered trial design that regulatory authorities now require. Addressing this interdependency directly, rather than treating each obstacle as a discrete engineering challenge, is likely to constitute the next phase of translational research in oncolytic virotherapy.

## Future directions and perspectives

**Fig. 4:**
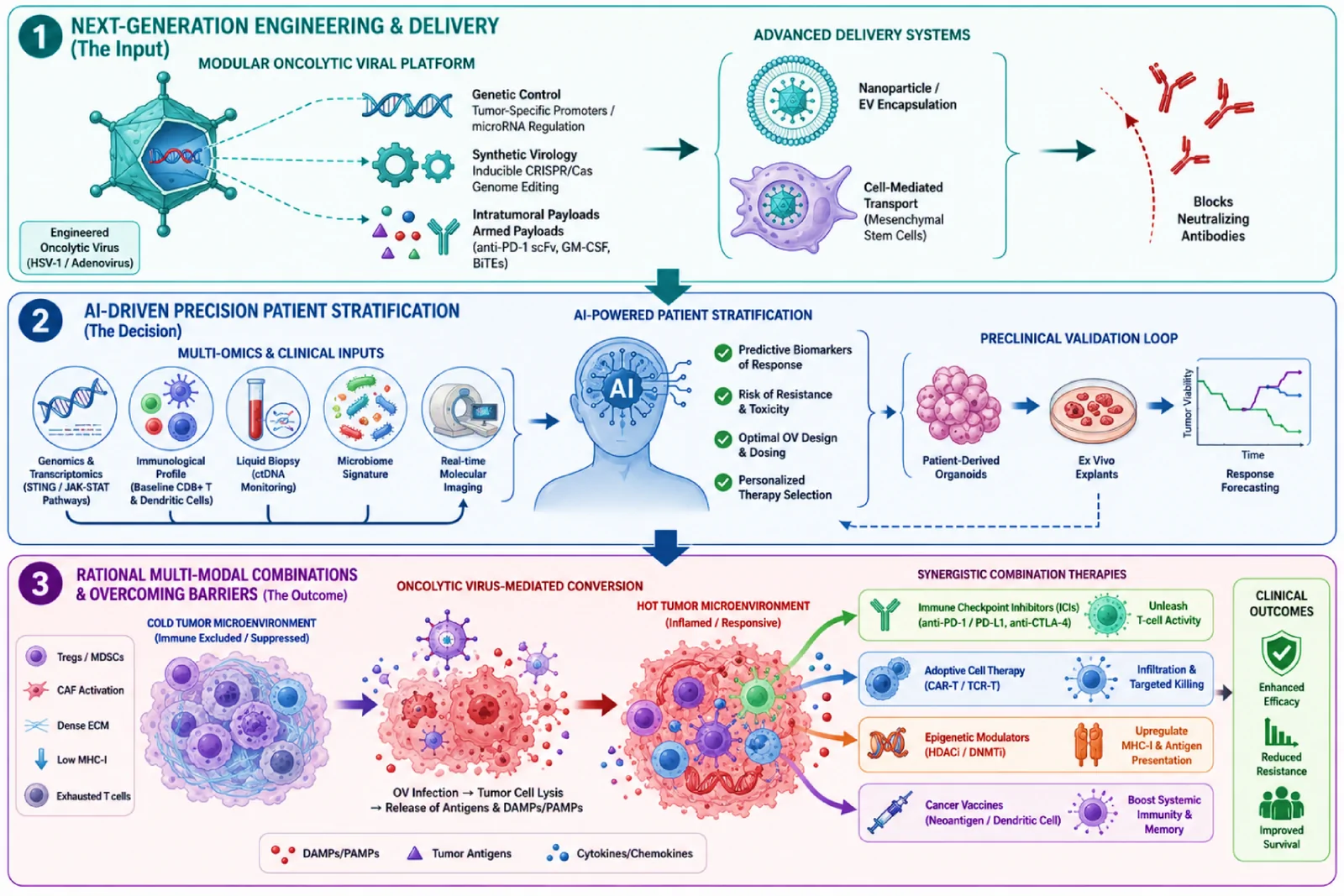
Next-generation engineering, precision stratification, and rational combinations shaping the future of oncolytic virotherapy. The paradigm is shifting away from standard viral monotherapies toward a personalized, multi-pronged precision oncology workflow. **(Top)** Next-generation synthetic engineering constructs modular viral platforms armed with localized immunomodulatory payloads (e.g., anti-PD-1 scFv, BiTEs, cytokines) and utilizes cellular/nanoparticle carriers to bypass systemic neutralizing antibodies. **(Middle)** Artificial intelligence and multi-omics data streams (genomics, transcriptomics, immune profiling, ctDNA, and patient-derived organoids) are integrated to generate multidimensional biomarker signatures for precise patient selection. **(Bottom)** The personalized oncolytic platform acts as an immune-stimulating backbone, converting immunologically "cold" microenvironments into "hot," highly inflamed lesions. This synergizes with rational, multi-modal combinations including checkpoint blockade, adoptive cell transfers, cancer vaccines, and epigenetic modulators to drive durable systemic antitumor immunity

The next phase of oncolytic virotherapy is expected to be driven less by the discovery of new viral species and more by advances in viral engineering, precision medicine, and rational combination therapy. Although substantial progress has been achieved over the past decade, current evidence indicates that durable clinical success will depend on matching viral design to tumor biology, immune context, and patient-specific characteristics rather than simply increasing viral complexity [16, 194]. Consequently, the field is shifting from developing universally applicable oncolytic viruses toward creating programmable therapeutic platforms tailored to individual tumors and integrated within broader precision oncology strategies (Fig. 4).

### Next-generation engineering of oncolytic viruses

Genetic engineering has become the major driving force behind the evolution of oncolytic virotherapy. Early-generation viruses relied primarily on their natural tumor tropism or simple attenuation, whereas next-generation platforms are designed as multifunctional therapeutics capable of simultaneously mediating tumor-selective replication, immune modulation, targeted payload delivery, and compatibility with combination therapies. This transition has substantially expanded the therapeutic potential of oncolytic viruses beyond direct tumor lysis [11, 195].

Current engineering strategies aim to maximize tumor specificity while minimizing toxicity to normal tissues. These approaches include tumor-specific promoters, microRNA-mediated regulation, receptor retargeting, controlled viral replication, and selective deletion of viral virulence genes [16]. Advances in synthetic virology are further broadening the design space by enabling modular viral platforms with inducible gene expression, programmable payload release, and the potential incorporation of genome-editing technologies such as CRISPR/Cas systems [196]. Such innovations offer unprecedented flexibility for developing viruses that respond dynamically to the tumor microenvironment while maintaining a favorable safety profile.

One of the most promising directions is the development of armed oncolytic viruses that deliver therapeutic molecules directly within tumors. Local expression of cytokines, chemokines, immune checkpoint inhibitors, bispecific T-cell engagers, costimulatory ligands, and suicide genes can substantially enhance intratumoral immune activation while reducing the systemic toxicity associated with conventional immunotherapy [11, 14, 16]. The clinical success of GM-CSF-expressing T-VEC established the proof of principle for this strategy, and newer viral constructs are now incorporating multiple immunomodulatory payloads to generate broader and more durable immune responses. For example, engineered HSV-1 vectors capable of selectively targeting epidermal growth factor receptor (EGFR)-expressing tumor cells while simultaneously producing functional anti-PD-1 single-chain antibodies illustrate how precision targeting and immune checkpoint blockade can be integrated within a single therapeutic platform [95, 197].

Improving systemic delivery remains another major engineering challenge. Although intratumoral administration has demonstrated encouraging efficacy in accessible lesions, many patients present with disseminated metastatic disease that requires effective systemic treatment. However, rapid viral neutralization by circulating antibodies, limited intratumoral penetration, abnormal tumor vasculature, and dense extracellular matrix components continue to restrict therapeutic efficacy. To overcome these barriers, considerable effort is being devoted to developing nanoparticle-based delivery systems, cell-mediated transport using mesenchymal stem cells or immune cells, magnetic nanoparticles, extracellular vesicle carriers, image-guided administration, and alternative delivery routes, including intraperitoneal and inhalational approaches [16, 22]. While many of these technologies remain at the preclinical or early clinical stage, they are expected to substantially improve viral biodistribution and therapeutic efficiency.

### Toward personalized oncolytic virotherapy

The future of oncolytic virotherapy is increasingly intertwined with precision oncology. Rather than treating all tumors with a single viral platform, growing evidence suggests that therapeutic success depends on selecting the most appropriate virus for each patient's unique tumor biology and immune landscape. Consequently, personalized virotherapy is becoming as much a challenge of patient selection as of viral engineering [198, 199].

Both tumor-intrinsic and host-related factors influence therapeutic responsiveness. Tumor permissiveness for viral replication, antiviral signaling defects, expression of viral entry receptors, antigen-presentation capacity, interferon pathway activity, and the composition of the tumor immune microenvironment all contribute to treatment outcome [16]. Host characteristics, including pre-existing antiviral immunity, innate immune responsiveness, previous viral exposure, and potentially the intestinal microbiome, may further modify viral persistence and antitumor activity. These observations indicate that no single biomarker is likely to accurately predict treatment response across all viral platforms or tumor types [11, 200]

Instead, future patient stratification will probably rely on multidimensional biomarker signatures that integrate genomic, transcriptomic, proteomic, immunological, and imaging data. Candidate biomarkers include PD-L1 expression, tumor mutational burden, defects in the STING or JAK–STAT signaling pathways, baseline infiltration by CD8⁺ T cells and dendritic cells, major histocompatibility complex class I expression, β2-microglobulin status, circulating tumor DNA, immune-related gene-expression signatures, and real-time molecular imaging of viral distribution [201, 202]. Integration of these complementary biomarkers is expected to improve patient selection, optimize therapeutic sequencing, and facilitate early identification of treatment resistance.

More predictive preclinical models will also be essential for advancing personalized virotherapy. Traditional cancer cell lines frequently fail to reproduce the complexity of human tumors and their surrounding immune microenvironment. Consequently, patient-derived organoids, patient-derived xenografts, ex vivo tumor explants, and humanized mouse models are increasingly being used to evaluate viral susceptibility and predict therapeutic response before clinical application [203, 204]. At the clinical level, adaptive trial designs incorporating longitudinal immune monitoring and biomarker-guided treatment modification may enable more efficient evaluation of novel viral platforms while reducing exposure to ineffective therapies.

The integration of artificial intelligence, machine learning, and multi-omics technologies is expected to further accelerate this transition toward precision virotherapy. Computational approaches capable of integrating genomic, immunological, radiological, and clinical data may facilitate patient stratification, optimize viral engineering, identify predictive biomarkers, and guide individualized combination strategies [205]. Although significant challenges related to cost, data standardization, regulatory approval, and clinical implementation remain, these technologies are likely to become increasingly important components of future oncolytic virotherapy.

### Rational combination therapies

Perhaps the most important conceptual shift in the field is the recognition that oncolytic viruses should be viewed primarily as immunotherapy platforms rather than stand-alone anticancer agents. Clinical experience has consistently demonstrated that viral monotherapy often produces limited and variable responses, whereas combination strategies can exploit the complementary mechanisms of multiple treatment modalities [206].

Future clinical development is therefore expected to focus increasingly on rational combinations with immune checkpoint inhibitors, adoptive cell therapies, cancer vaccines, radiotherapy, chemotherapy, targeted therapies, and epigenetic modulators [207]. Viral infection can enhance antigen presentation, promote immune-cell recruitment, reverse local immunosuppression, and convert immunologically "cold" tumors into inflamed, treatment-responsive lesions, thereby creating favorable conditions for subsequent immunotherapy [11, 202]. The challenge now lies not only in identifying effective combinations but also in determining the optimal treatment sequence, dosing schedule, route of administration, and patient population for each therapeutic strategy [11, 117].

### Clinical translation and regulatory perspectives

Despite encouraging advances, several obstacles continue to slow the widespread clinical implementation of oncolytic virotherapy. Manufacturing large quantities of genetically engineered viruses under Good Manufacturing Practice (GMP) conditions remains technically demanding and costly. Regulatory approval is further complicated by the biological complexity of replication-competent viruses, long-term biosafety considerations, and the need to evaluate both antiviral and antitumor immune responses. In addition, greater international harmonization of regulatory frameworks and standardized clinical endpoints will be essential to facilitate comparison among clinical trials and accelerate therapeutic development [208].

Future clinical studies are also expected to incorporate biomarker-driven patient selection, longitudinal immune monitoring, adaptive trial designs, and standardized translational endpoints. Such approaches should improve trial efficiency while providing mechanistic insight into treatment response and resistance. Together with continued advances in viral engineering and precision oncology, these strategies are expected to facilitate the transition of oncolytic virotherapy from experimental treatment to routine clinical practice [16, 201].

## Conclusion

Oncolytic virotherapy has evolved from an intriguing biological observation into one of the most promising platforms in modern cancer immunotherapy. Advances in molecular virology, synthetic biology, and immuno-oncology have transformed naturally occurring viruses into programmable therapeutic agents capable of selectively eliminating malignant cells while simultaneously stimulating potent local and systemic antitumor immune responses. These developments have fundamentally changed the perception of oncolytic viruses from simple tumor-lytic agents to multifunctional immunotherapeutic platforms that actively remodel the tumor microenvironment and enhance the efficacy of other anticancer therapies.

Clinical translation has already demonstrated the feasibility and safety of this therapeutic approach, culminating in regulatory approval of several oncolytic viruses and a rapidly expanding portfolio of engineered viral platforms. Nevertheless, important challenges remain, including efficient systemic delivery, antiviral immune clearance, tumor heterogeneity, biomarker identification, manufacturing complexity, and optimization of combination strategies. Addressing these limitations will require continued collaboration among virologists, immunologists, oncologists, bioengineers, computational scientists, and regulatory agencies.

Looking forward, the future of oncolytic virotherapy will likely be defined not by increasingly complex viruses alone but by the integration of advanced viral engineering with precision oncology. Biomarker-guided patient selection, multifunctional viral platforms, innovative delivery technologies, artificial intelligence-assisted therapeutic design, and rational combination regimens are expected to transform oncolytic viruses into highly personalized cancer therapeutics. As these technologies mature and clinical evidence continues to accumulate, oncolytic virotherapy is poised to become an integral component of personalized immuno-oncology, offering more effective and durable treatment options for carefully selected patients across a broad spectrum of malignancies.

## Data Availability

Not Applicable.

## Abbreviations

AAV: Adeno-associated virus
APC: Antigen-presenting cell
ATP: Adenosine triphosphate
BCMA: B-cell maturation antigen
CAR: Coxsackievirus and adenovirus receptor
CAR-T: Chimeric antigen receptor T cell
CCL5: C–C motif chemokine ligand 5
cfDNA: Cell-free DNA
cGAS: Cyclic GMP–AMP synthase
CR: Complete response
CRAds: Conditionally replicating adenoviruses
CRT: Calreticulin
CTLA-4: Cytotoxic T-lymphocyte-associated protein 4
DAMPs: Damage-associated molecular patterns
DNA: Deoxyribonucleic acid
DRR: Durable response rate
DSG2: Desmoglein-2
EGFR: Epidermal growth factor receptor
EMA: European Medicines Agency
GM-CSF: Granulocyte–macrophage colony-stimulating factor
HIF-1: Hypoxia-inducible factor-1
HMGB1: High mobility group box 1
HSP70: Heat shock protein 70
HSP90: Heat shock protein 90
HSPs: Heat shock proteins
HSV: Herpes simplex virus
HSV-1: Herpes simplex virus type 1
HVEM: Herpesvirus entry mediator
ICAM-1: Intercellular adhesion molecule−1
ICD: Immunogenic cell death
ICI: Immune checkpoint inhibitor
IFN: Interferon
IFNAR: Interferon-α/β receptor
IL: Interleukin
IL-7: Interleukin-7
IL-12: Interleukin-12
IL-15: Interleukin-15
IL-21: Interleukin-21
JNK: C-Jun N-terminal kinase
LRP1: Low-density lipoprotein receptor-related protein 1
MDA5: Melanoma differentiation-associated protein 5
MHC: Major histocompatibility complex
MLKL: Mixed lineage kinase domain-like protein
MSCs: Mesenchymal stromal cells
MV: Measles virus
NDV: Newcastle disease virus
NK: Natural killer
NLRP3: NOD-like receptor family pyrin domain-containing 3
ORR: Overall response rate
OS: Overall survival
OV: Oncolytic virus
OVs: Oncolytic viruses
OVT: Oncolytic virotherapy
PAMPs: Pathogen-associated molecular patterns
PD-1: Programmed cell death protein 1
PD-L1: Programmed death-ligand 1
PFS: Progression-free survival
PI3K: Phosphoinositide 3-kinase
PKR: Protein kinase R
RAGE: Receptor for advanced glycation end products
RAS: Rat sarcoma proto-oncogene
RB: Retinoblastoma protein
RCD: Regulated cell death
RIG-I: Retinoic acid-inducible gene I
RIPK1: Receptor-interacting protein kinase 1
RIPK3: Receptor-interacting protein kinase 3
RNA: Ribonucleic acid
ROS: Reactive oxygen species
SFV: Semliki Forest virus
SLAM: Signaling lymphocytic activation molecule
SMAC: Second mitochondria-derived activator of caspases
STING: Stimulator of interferon genes
TAAs: Tumor-associated antigens
TLR: Toll-like receptor
TLR4: Toll-like receptor 4
TME: Tumor microenvironment
TNF-R: Tumor necrosis factor receptor
TRAIL: TNF-related apoptosis-inducing ligand
TRAIL-R: TNF-related apoptosis-inducing ligand receptor
T-VEC: Talimogene laherparepvec
uPAR: Urokinase-type plasminogen activator receptor
VSV: Vesicular stomatitis virus
VV: Vaccinia virus
Wnt: Wingless/Integrated signaling pathway

## References

[1] Elbarazi I, Ahmed LA. Alleviating the global burden of cancer through prevention and early detection. Cancer Control. 2025;32:1–4. 10.1177/10732748251378666. PMC12432317 40939582

[2] C. M. Balch et al., “The global cancer crisis : a review of growing burden , deepening inequality and initiatives for prevention and early detection,” *Ecancermedicalscience*, pp. 1–23, 2026. 10.3332/ecancer.2026.2071 PMC13006544 41877745

[3] Zafar A, Khatoon S, Jawad M, Junaid K, Aisha A. Advancements and limitations in traditional anti - cancer therapies: a comprehensive review of surgery, chemotherapy, radiation therapy, and hormonal therapy. Discov Oncol. 2025. 10.1007/s12672-025-02198-8. PMC12021777 40272602

[4] V. Murugesan and T. Thuraisingam, “From Excision to Immunity : The Full Spectrum of Modern Melanoma Treatments,” *Cancers (Basel).*, pp. 1–33, 2026. 10.3390/cancers18132043 PMC13359695 42449588

[5] Lovly CM, Salama AKS, Salgia R. Tumor heterogeneity and therapeutic resistance. Am Soc Clin Oncol Educ book Am Soc Clin Oncol Annu Meet. 2023. 10.1200/EDBK. PMC10132823 27249771

[6] H. Miao et al., “Transforming the landscape of cancer treatment with seven promising novel therapies: evolution and future perspectives,” *Med. Plus*, vol. 2, no. May, 2025, 10.1016/j.medp.2025.100087.

[7] Wang Y. Advances in cancer immunotherapy and future directions in personalized medicine. Open Life Sci. 2025. 10.1515/biol-2025-1179. PMC12596039 41211067

[8] Bandara S, Raveendran S. Current landscape and future directions in cancer immunotherapy: therapies, trials, and challenges. Cancers. 2025. 10.3390/cancers17050821. PMC11899461 40075668

[9] J. et al. Fan, T., Zhang, M., Yang, “Therapeutic cancer vaccines : advancements , challenges and prospects,” *Signal Transduct. Target. Ther.*, no. May, 2023, 10.1038/s41392-023-01674-3. PMC10716479 38086815

[10] Ashonibare VJ, et al. Current evidence and emerging approaches in immunotherapy for gynecological cancers. Discov Oncol. 2026. 10.1007/s12672-026-04785-9. PMC13149720 41896372

[11] Y. et al. Xiao, D., Zhang, H., Liu, “Oncolytic viruses : advanced strategies in cancer therapy,” *Sig Transduct Target Ther*, no. July, 2026. 10.1038/s41392-025-02343-3 PMC12873401 41639062

[12] G. Cao, C. Ding, J. Dai, and X. Qiu, “Oncolytic virus and immunogenic cell death in cancer therapy,” *Tumour Virus Res.*, vol. 20, no. July, 2025. 10.1016/j.tvr.2025.200333 PMC12718214 41314288

[13] G. Bell, J., & McFadden, “Viruses for Tumor Therapy,” *Cell Host Microbe*, vol. 15, no. 3, pp. 260–265, 2015, 10.1016/j.chom.2014.01.002.Viruses. PMC3963258 24629333

[14] Zheng M, Huang J, Tong A, Yang H. Oncolytic viruses for cancer therapy: barriers and recent advances. Mol Ther Oncolytics. 2019;15(37):234–47. 10.1016/j.omto.2019.10.007. PMC6911943 31872046

[15] A. Jadhav and J. Aravind, “A comprehensive review redefining viruses as therapeutic agents in cancer treatment,” *Discov. Oncol.*, 2025. 10.1007/s12672-025-03973-3 PMC12753620 41313526

[16] Fayyad-kazan M, Al-tameemi S. Oncolytic viruses in cancer immunotherapy: from molecular engineering to clinical translation. Cells. 2026. 10.3390/cells15050393. PMC12984100 41827827

[17] Volovat SR, et al. Oncolytic virotherapy: a new paradigm in cancer immunotherapy. Int J Mol Sci. 2024. 10.3390/ijms25021180. PMC10816814 38256250

[18] D. Lin, Y. Shen, and T. Liang, “Oncolytic virotherapy : basic principles , recent advances and future directions,” *Sig Transduct Target Ther*, no. October 2022, 2023, 10.1038/s41392-023-01407-6. PMC10090134 37041165

[19] Alam SMS, Tasnim M, Amin T, Islam SMBU. A new era in oncology: clinical insights into the application of oncolytic viruses. Cancer Control. 2025;32:1–20. 10.1177/10732748251382295. PMC12461052 40991899

[20] Johnson DB, Puzanov I, Kelley MC. Talimogene laherparepvec (T-VEC) for the treatment of advanced melanoma. Immunotherapy. 2016;7(6):611–9. 10.2217/imt.15.35.Talimogene. PMC4519012 26098919

[21] Erickson NJ, Stavarache M, Tekedereli I, Kaplitt MG, Markert JM. Herpes Simplex Oncolytic Viral Therapy for Malignant Glioma and Mechanisms of Delivery. World Neurosurg. 2025;194:123595. 10.1016/j.wneu.2024.123595. PMC12094189 39710201

[22] Rivera S, Jhommara O, Palacios D, Sofía Z, Mosquera O. Oncolytic virotherapy and tumor microenvironment modulation. Clin Exp Med. 2025;25(1):1–12. 10.1007/s10238-025-01691-2. PMC12277230 40685482

[23] Chen C, et al. Oncolytic virotherapy in solid tumors: a current review. BioDrugs. 2025;39(6):857–76. 10.1007/s40259-025-00743-z. PMC12568882 40993465

[24] S. E. Berkey, S. H. Thorne, and D. L. Bartlett, “Oncolytic Virotherapy and the Tumor Microenvironment,” *Adv. Exp. Med. Biol. vol 1036. Springer, Cham*, 2017. 10.1007/978-3-319-67577-0_11 29275471

[25] Chen L, Zuo M, Zhou Q, Wang Y. Oncolytic virotherapy in cancer treatment: challenges and optimization prospects. Front Immunol. 2023;December:1–14. 10.3389/fimmu.2023.1308890. PMC10758479 38169820

[26] Y. Li, X. Qin, C. Liang, and L. Wang, “Progress in the research and development of oncolytic virus therapies,” *Front. Pharmacol.*, no. February, pp. 1–14, 2026, 10.3389/fphar.2026.1751206. PMC12960624 41800095

[27] Russell SJ, Bell JC, Engeland CE, McFadden G. Advances in oncolytic virotherapy. Commun Med. 2022. 10.1038/s43856-022-00098-4. PMC9053215 35603308

[28] Hua X, et al. Bridging mechanism and clinic: unlocking the full potential of oncolytic virus-based immunotherapy. Mol Cancer. 2026. 10.1186/s12943-026-02651-4. PMC13162530 41917920

[29] Y. Guo, C. Huang, L. Xu, Y. Xuan, and P. Wang, “Advances in oncolytic virus delivery strategies and challenges in clinical translation,” *Mater. Today Bio*, vol. 39, no. May, p. 103316, 2026, 10.1016/j.mtbio.2026.103316. PMC13273587 42317522

[30] Lawler SE, Speranza M, Cho C, Chiocca EA. Oncolytic viruses in cancer treatment a review. JAMA Oncol. 2017. 10.1001/jamaoncol.2016.2064. 27441411

[31] Chouljenko DV, Ding J, Lee I, Murad YM, Bu X, Liu G. Induction of durable antitumor response by a novel oncolytic herpesvirus expressing multiple immunomodulatory transgenes. Biomedicines. 2020. 10.3390/biomedicines8110484. PMC7695276 33182232

[32] Davola ME, Mossman KL. Oncolytic viruses : how ‘ lytic ’ must they be for therapeutic efficacy ? Oncolytic viruses : how ‘ lytic ’ must they be for therapeutic efficacy ? Oncoimmunology. 2019;8(6):1–7. 10.1080/2162402X.2019.1596006. PMC6492965 31069150

[33] Wang L, Dunmall LSC, Cheng Z, Wang Y. Remodeling the tumor microenvironment by oncolytic viruses: beyond oncolysis of tumor cells for cancer treatment. J Immunother Cancer. 2022. 10.1136/jitc-2021-004167. PMC9157365 35640930

[34] Alwithenani A, Hengswat P, Chiocca EA. Oncolytic viruses as cancer therapeutics : From mechanistic insights to clinical translation. Mol Ther. 2025;33(5):2217–28. 10.1016/j.ymthe.2025.03.035. PMC12126799 40143547

[35] H. Qazi, T. J., Nunes, A. T., Xavier, P., & Fukumasu, “Oncolytic virotherapy: molecular mechanisms, delivery strategies, and translational insights,” *Crit. Rev. Oncol. Hematol.*, 2025, doi: . 10.1016/j.critrevonc.2025.105061. 41325798

[36] Guo ZS, Liu Z, Bartlett DL. Oncolytic immunotherapy : dying the right way is a key to eliciting potent antitumor immunity. Front Oncol. 2014;4(April):1–11. 10.3389/fonc.2014.00074. PMC3989763 24782985

[37] Xu J, Xia Z, Wang S, Xia Q. Resistance to oncolytic virotherapy: multidimensional mechanisms and therapeutic breakthroughs (review). Int J Mol Med. 2025. 10.3892/ijmm.2025.5612. PMC12404896 40849827

[38] Enow JA, Sheikh HI, Rahman MM. Tumor tropism of DNA viruses for oncolytic virotherapy. Viruses. 2023. 10.3390/v15112262. PMC10675630 38005938

[39] Apolonio JS, et al. Oncolytic virus therapy in cancer: a current review. World J Virol. 2021;10(5):229–55. 10.5501/wjv.v10.i5.229. PMC8474975 34631474

[40] Xu J, Liu C, An Y, Sun J, Wang S, Xia Q. Mechanisms of oncolytic virus-induced multi-modal cell death and therapeutic prospects. Int J Mol Sci. 2025. 10.3390/ijms26199770. PMC12524531 41097035

[41] Inoue H. Oncolytic virotherapy and immunogenic cell death : mechanisms , platforms , and clinical translation. Viruses. 2026. 10.3390/v18040461. PMC13120409 42043250

[42] Moaven O, Mangieri CW, Stauffer JA, Anastasiadis PZ, Borad MJ. Strategies to develop potent oncolytic viruses and enhance their therapeutic efficacy. JCO Precis Oncol. 2021;1:733–43. 10.1200/PO.21.00003 PMC8232397 34250395

[43] Ehrlich M, Bacharach E. Oncolytic virotherapy: the cancer cell side. Cancers (Basel). 2021. 10.3390/cancers13050939. PMC7956656 33668131

[44] Zhang B, Cheng P. Improving antitumor efficacy via combinatorial regimens of oncolytic virotherapy. Mol Cancer. 2020(1). 10.1186/s12943-020-01275-6. PMC7656670 33172438

[45] Y. Chen et al., “Current status and research progress of oncolytic virus,” *Pharm. Sci. Adv.*, vol. 2, no. November 2023, p. 100037, 2024, 10.1016/j.pscia.2024.100037. PMC12710064 41550155

[46] Q. Fernandes and O. Gbenga, “Cytokine Unveiling the nexus : The tumor microenvironment as a strategic frontier in viral cancers,” *Cytokine*, vol. 185, no. September 2024, p. 156827, 2025, 10.1016/j.cyto.2024.156827. 39647395

[47] Bai Y, Hui P, Du X, Su X. Updates to the antitumor mechanism of oncolytic virus. Thorac Cancer. 2019;10:1031–5. 10.1111/1759-7714.13043. PMC6501037 30900824

[48] Inoue H, Tani K. Multimodal immunogenic cancer cell death as a consequence of anticancer cytotoxic treatments. Cell Death Differ. 2014. 10.1038/cdd.2013.84. PMC3857623 23832118

[49] Verheije MH, Rottier PJM. Retargeting of viruses to generate oncolytic agents. Adv Virol. 2012. 10.1155/2012/798526. PMC3265223 22312365

[50] Blackwell JL, et al. Retargeting to EGFR enhances adenovirus infection efficiency of squamous cell carcinoma. Arch Otolaryngol Head Neck Surg. 1999;125:856–63. 10.1001/archotol.125.8.856 10448731

[51] Sakurai F, Nishimae F, Takayama K, Mizuguchi H. Optimization of an E1A gene expression cassette in an oncolytic adenovirus for efficient tumor cell killing activity. Anticancer Res. 2021;782:773–82. 10.21873/anticanres.14829. 33517282

[52] C. Larson, B. Oronsky, J. Scicinski, G. R. Fanger, A. Oronsky, and T. R. Reid, “Going viral : a review of oncolytic adenoviruses,” *Oncotarget*, vol. 6, no. 24, 2015. 10.18632/oncotarget.5116 PMC4652981 26280277

[53] Geisler A, Fechner H. MicroRNA-regulated viral vectors for gene therapy. World J Exp Med. 2016;6(2):37–55. 10.5493/wjem.v6.i2.37. PMC4873559 27226955

[54] Dhungel JC, Ramlogan-Steel CA, Steel B. MicroRNA-regulated gene delivery systems for research and therapeutic purposes. Molecules. 2018. 10.3390/molecules23071500. PMC6099389 29933586

[55] Yoon A, Rivera-cruz C, Gimble JM, Yun C, Figueiredo ML. Immunotherapy by mesenchymal stromal cell delivery of oncolytic viruses for treating metastatic tumors. Mol Ther Oncolytics. 2022;25(June):78–97. 10.1016/j.omto.2022.03.008. PMC8989711 35434272

[56] Xia Y, Li D, Yang K, Ou X. Systemic delivery strategies for oncolytic viruses: advancing targeted and efficient tumor therapy. Int J Mol Sci. 2025. 10.3390/ijms26146900. PMC12295215 40725148

[57] V. A. Sarkisova, A. A. Dalina, D. O. Neymysheva, M. A. Zenov, G. V Ilyinskaya, and P. M. Chumakov, “Cell Carriers for Oncolytic Virus Delivery : Prospects for Systemic Administration,” *Cancers (Basel).*, pp. 1–25, 2025. 10.3390/cancers17142296 PMC12293357 40723180

[58] Aljabali AAA, Bashatwah R, Gammoh O. The dual promise of oncolytic viruses: selective targeting and therapeutic enhancement in cancer treatment. Explor Immunol. 2025. 10.37349/ei.2025.1003204.

[59] Isaeva AS, et al. Shaping viral immunotherapy towards cancer-targeted immunological cell death. Front Oncol. 2025. 10.3389/fonc.2025.1540397. PMC12280590 40697381

[60] Kortum F, Hartmann N, Bryan A, Glau S, Altomonte J. Syncytial death mediated by oncolytic rVSV-NDV dynamically activates immunogenic apoptosis and necroptosis in human lung cancer cells. Mol Ther Oncol. 2025;33(September):1–21. 10.1016/j.omton.2025.201027. PMC12390950 40896365

[61] Takasu A, Masui A, Hamada M, Imai T, Iwai S, Yura Y. Immunogenic cell death by oncolytic herpes simplex virus type 1 in squamous cell carcinoma cells. Cancer Gene Ther. 2016. 10.1038/cgt.2016.8. 26987291

[62] L. Palanivelu and C. Liu, “Immunogenic cell death: The cornerstone of oncolytic viro-immunotherapy,” *Front. Immunol.*, no. January, pp. 1–13, 2023, 10.3389/fimmu.2022.1038226. PMC9899992 36755812

[63] H. Tazawa, S. Kuroda, J. Hasei, and S. Kagawa, “Impact of Autophagy in Oncolytic Adenoviral Therapy for Cancer,” *Int. J. Mol. Sci.*, pp. 1–13, 2017, 10.3390/ijms18071479. PMC5535969 28698504

[64] H. S. Rodriguez-Rocha, H., Gomez-Gutierrez, J. G., Garcia-Garcia, A., Rao, X. M., Chen, L., McMasters, K. M., & Zhou, “Adenoviruses induce autophagy to promote virus replication and oncolysis,” *Virology*, vol. 416, no. 502, pp. 9–15, 2011, 10.1016/j.virol.2011.04.017.Adenoviruses. PMC3113480 21575980

[65] K. M. Calvillo-rodr and A. C. Mart, “Immunotherapies inducing immunogenic cell death in cancer : insight of the innate immune system,” *Front. Immunol.*, no. November, pp. 1–21, 2023, 10.3389/fimmu.2023.1294434. PMC10701401 38077402

[66] Y. Wu, T. Sun, M. Chen, M. Munir, and H. Liu, “Oncolytic viruses-modulated immunogenic cell death , apoptosis and autophagy linking to virotherapy and cancer immune response,” *Front. Cell. Infect. Microbiol.*, no. March, pp. 1–13, 2023, 10.3389/fcimb.2023.1142172. PMC10050605 37009515

[67] Nailwal H, Chan FK. Necroptosis in anti-viral inflammation. Cell Death Differ. 2018. 10.1038/s41418-018-0172-x. PMC6294789 30050058

[68] Zhang Z, et al. Induced necroptosis and its role in cancer immunotherapy. Int J Mol Sci. 2024. 10.3390/ijms251910760. PMC11477008 39409087

[69] T. Kuriakose and T. Kanneganti, “Pyroptosis in Antiviral Immunity,” *Curr. Top. Microbiol. Immunol.*, no. 901, 2023, doi:10.1007/82 10.1007/82_2019_189 PMC7314647 31875268

[70] Chen H, et al. Advances in the mechanism of inflammasomes activation in herpes virus infection. Front Immunol. 2024. 10.3389/fimmu.2024.1346878. PMC10999540 38590522

[71] Alifano M, Cremer I, Leonardi L, Sibe S, Albanese C, Romero-garcia S. Autophagy modulation by viral infections influences tumor development. Front Oncol. 2021;11(October):1–19. 10.3389/fonc.2021.743780. PMC8569469 34745965

[72] Jiang H, White EJ, Ríos-vicil CI, Xu J, Gomez-manzano C, Fueyo J. Human adenovirus type 5 induces cell lysis through autophagy and autophagy-triggered caspase activity. J Virol. 2011;85(10):4720–9. 10.1128/JVI.02032-10. PMC3126198 21367888

[73] Zhang L, et al. Autophagy induced by human adenovirus B7 structural protein VI inhibits viral replication. Virol Sin. 2023;38(February):709–22. 10.1016/j.virs.2023.08.002. PMC10590704 37549881

[74] C. Kan, X., Yin, Y., Song, C., Tan, L., Qiu, X., Liao, Y., Liu, W., Meng, S., Sun, Y., & Ding, “Newcastle-disease-virus-induced ferroptosis through nutrient deprivation and ferritinophagy in tumor cells,” *ISCIENCE*, vol. 24, no. 8, p. 102837, 2021, 10.1016/j.isci.2021.102837. PMC8326413 34368653

[75] Sun Y, et al. Oncolytic Newcastle disease virus induced degradation of YAP through E3 ubiquitin ligase PRKN to exacerbate ferroptosis in tumor cells. J Virol. 2024. 10.1128/jvi.01897-23. PMC10949840 38411946

[76] Ghavimi R, et al. Viral warriors: unlocking the immune system’s potential with oncolytic viruses in cancer immunotherapy. Mol Ther Oncolytics. 2025;33(4):201100. 10.1016/j.omton.2025.201100. PMC12721169 41438130

[77] Ahmed A, Tait SWG. Targeting immunogenic cell death in cancer. Mol Oncol. 2020;14:2994–3006. 10.1002/1878-0261.12851. PMC7718954 33179413

[78] Mardi A, et al. Biological causes of immunogenic cancer cell death (ICD) and anti-tumor therapy; combination of oncolytic virus-based immunotherapy and CAR T-cell therapy for ICD induction. Cancer Cell Int. 2022. 10.1186/s12935-022-02585-z. PMC9052538 35488303

[79] Schcolnik A, Bernardo C, Mandy O, Mayra J, Rivera C, Flisser A. Calreticulin in phagocytosis and cancer: opposite roles in immune response outcomes. Apoptosis. 2019;24(3):245–55. 10.1007/s10495-019-01532-0. 30929105

[80] M. Kielbik, I. Szulc-kielbik, and M. Klink, “Calreticulin — Multifunctional Chaperone in Immunogenic Cell Ovarian Cancer Patients,” *Cells*, 2021. 10.3390/cells10010130 PMC7827772 33440842

[81] Y. Kim, D. R. Clements, A. M. Sterea, H. W. Jang, S. A. Gujar, and P. W. K. Lee, “Dendritic Cells in Oncolytic Virus-Based Anti-Cancer Therapy,” *Viruses*, pp. 6506–6525, 2015, 10.3390/v7122953. PMC4690876 26690204

[82] and T. D. Bhatt, Darshak K., “Molecular Circuits of Immune Sensing and Response to Oncolytic Virotherapy,” *Int. J. Mol. Sci.*, 2024. 10.3390/ijms25094691 PMC11083234 38731910

[83] Nelson KL, et al. VAX014 activates tumor-intrinsic STING and RIG-I to promote the development of antitumor immunity. Mol Cancer Ther. 2025;23:587–604. 10.1158/1535-7163.MCT-24-0509. PMC11962400 39868467

[84] C. F. Kollmann, N. Van Montfoort, P. Cordelier, J. Pol, and D. Olagnier, “Oncolytic virotherapy : Sparking durable anti-tumor immunity through microenvironment modulation,” *Semin. Immunol.*, vol. 80, no. August, p. 101994, 2025, 10.1016/j.smim.2025.101994. 40992319

[85] V. W. Nadafi, R., Dong, W., & Van Beusechem, “Immunological Impact of Oncolytic Adenoviruses On Cancer Therapy : Clinical Insights,” *Eur. J. Immunol.*, pp. 1–15, 2025, 10.1002/eji.70024. PMC12304604 40726054

[86] I. Barjij and M. Meliani, “Immunogenic Cell Death as a Target for Combination Therapies in Solid Tumors : A Systematic Review Toward a New Paradigm in Immuno-Oncology,” *Cureus*, vol. 17, no. 6, 2025, 10.7759/cureus.85776. PMC12248260 40656384

[87] F. Ju et al., “Oncolytic virus expressing PD- ­ inhibitors activates a collaborative intratumoral immune response to control tumor and synergizes with 3 blockade,” *J. Immunother. cancer*, pp. 1–17, 2022, 10.1136/jitc-2022-004762. PMC9189843 35688558

[88] De Matos AL, Franco LS, Mcfadden G. Oncolytic viruses and the immune system: the dynamic duo. Mol Ther Methods Clin Dev. 2020;17(June):349–58. 10.1016/j.omtm.2020.01.001. PMC7015832 32071927

[89] Al-obaidi I, Sandhu C, Qureshi B, Seymour LW. The implications of oncolytic viruses targeting fibroblasts in enhancing the antitumoural immune response. Heliyon. 2024;10(20):e39204. 10.1016/j.heliyon.2024.e39204. PMC11535324 39502212

[90] Zubair A, Lazarou L, Alkahtani AM, Shahani MY, Afghan N. Enhancing antitumor immunity through oncolytic viruses: advances in genetic engineering. Discov Oncol. 2026. 10.1007/s12672-026-04792-w. PMC13090473 41811580

[91] T. P. Kaur, B., Chiocca, E. A., & Cripe, “Oncolytic HSV-1 Virotherapy: Clinical Experience and Opportunities for Progress,” *Curr. Pharm. Biotechnol.*, vol. 13, no. 9, pp. 1842–1851, 2014. 10.2174/138920112800958814 PMC4233127 21740359

[92] Scanlan H, Coffman Z, Bettencourt J, Shipley T, Bramblett DE. Herpes simplex virus 1 as an oncolytic viral therapy for refractory cancers. Front Oncol. 2022;July:1–18. 10.3389/fonc.2022.940019. PMC9364694 35965554

[93] Zhou M, Shen Z. Advanced progress in the genetic modification of the oncolytic HSV-1 virus. Front Oncol. 2025. 10.3389/fonc.2024.1525940. PMC11790444 39906660

[94] Martuza RL. Conditionally replicating herpes vectors for cancer therapy. J Clin Invest. 2000;105(7):841–6. 10.1172/JCI9744 PMC382984 10749560

[95] Epstein AL, Rabkin SD. Safety of Non-Replicative and Oncolytic Replication-Selective HSV Vectors. Trends Mol Med. 2025;30(8):781–94. 10.1016/j.molmed.2024.05.014.Safety. PMC11329358 38886138

[96] Zager JS, Delman KA, Weigel TL, Rusch VW. Replication-competent herpes virus NV1020 as direct treatment of pleural cancer in a rat model. J Thorac Cardiovasc Surg. 2002. 10.1067/mtc.2002.122297. 12091817

[97] Chahlavi A, Todo T, Martuza RL, Rabkin SD, Dc W. Replication-competent herpes simplex virus vector G207 and cisplatin combination therapy for head and neck squamous cell carcinoma. Neoplasia. 1999;1(2):162–9. 10.1038/sj.neo.7900016 PMC1508135 10933051

[98] Mochizuki AY, et al. Intratumoral / peritumoral herpes simplex virus-1 mutant HSV1716 in pediatric patients with refractory or recurrent high-grade gliomas : a report of the Pediatric Brain Tumor Consortium. Onco. 2025. 10.3390/onco5010001.

[99] T. Todo et al., “Intratumoral oncolytic herpes virus G47∆ for residual or recurrent glioblastoma: a phase 2 trial,” *Nat. Med.*, vol. 28, no. August, 2022, 10.1038/s41591-022-01897-x. PMC9388376 35864254

[100] P. B. Hajeri, N. S. Sharma, and M. Yamamoto, “Oncolytic Adenoviruses : Strategies for Improved Targeting and Specificity,” *Cancers (Basel).*, pp. 1–27, 2020. 10.3390/cancers12061504 PMC7352392 32526919

[101] Tan EW, Abd-aziz N, Poh CL. Engineered oncolytic adenoviruses: an emerging approach for cancer therapy. Pathogens (Basel). 2022. 10.3390/pathogens11101146. PMC9608483 36297203

[102] K. Wold, W. S., & Toth, “Adenovirus Vectors for Gene Therapy, Vaccination and Cancer Gene Therapy,” *Curr Gene Ther.*, vol. 13, no. 6, pp. 421–433, 2013. 10.2174/1566523213666131125095046 PMC4507798 24279313

[103] Oronsky B, Gastman B, Conley AP, Reid C, Caroen S, Reid T. Oncolytic adenoviruses: the cold war against cancer finally turns hot. Cancers (Basel). 2022. 10.3390/cancers14194701. PMC9562194 36230621

[104] Yao J, Shayakhmetov DM. Oncolytic adenoviruses in bladder and kidney cancers: emerging strategies and next frontiers. Front Microbiol. 2025. 10.3389/fmicb.2025.1729087. PMC12722901 41445946

[105] Tseha ST, David J. Role of Adenoviruses in Cancer Therapy. Front Oncol. 2022;12(June):1–17. 10.3389/fonc.2022.772659. PMC9218278 35756634

[106] Stawowczyk M, Ye Y, Chen NG. Vaccinia Virus — A Swiss Army Knife Against Cancer. Cancers (Basel). 2025;858:1–41. 10.3390/cancers17142324 PMC12293100 40723208

[107] Pelin A, Boulton S, Tamming LA, Bell JC. Engineering vaccinia virus as an immunotherapeutic battleship to overcome tumor heterogeneity. Expert Opin Biol Ther. 2020. 10.1080/14712598.2020.1757066. 32297534

[108] L. Xu, H. Sun, N. R. Lemoine, Y. Xuan, and P. Wang, “Oncolytic vaccinia virus and cancer immunotherapy,” *Front. Immunol.*, vol. 1, no. January, 2024, 10.3389/fimmu.2023.1324744. PMC10811104 38283361

[109] Deng L, et al. Oncolytic efficacy of thymidine kinase-deleted vaccinia virus. Oncotarget. 2017;8(25):40533–43. 10.18632/oncotarget.17125 PMC5522336 28465492

[110] Abou-alfa GK, Galle R, Chao Y. PHOCUS: a phase 3, randomized, open-label study of sequential treatment with Pexa-Vec (JX-594) and sorafenib in patients with advanced hepatocellular carcinoma. Liver Cancer. 2024. 10.1159/000533650. PMC11095598 38756145

[111] Cripe TP, et al. Phase 1 study of intratumoral Pexa-Vec ( JX-594), an oncolytic and immunotherapeutic vaccinia virus, in pediatric cancer patients. Mol Ther. 2015;23(3):602–8. 10.1038/mt.2014.243. PMC4351466 25531693

[112] Foloppe J, et al. TG6002 oncolytic vaccinia virus and chemotherapy synergy: a promising strategy for pancreatic ductal adenocarcinoma. Front Microbiol. 2025. 10.3389/fmicb.2025.1656984. PMC12506463 41070126

[113] Gong J, Sachdev E, Mita AC, Mita MM. Clinical development of reovirus for cancer therapy: an oncolytic virus with immune-mediated antitumor activity. World J Methodol. 2016;6(1):25–42. 10.5662/wjm.v6.i1.25. PMC4804250 27019795

[114] Alain T, et al. Reovirus therapy of lymphoid malignancies. Neoplasia. 2002;100(12):4146–53. 10.1182/blood-2002-02-0503. 12393565

[115] Kim SY, Maitra R, Goel S. Multimodal immune activation abilities and characteristics of reovirus. Am J Transl Res. 2021;13(12):14176–85. PMC8748157 35035763

[116] Sharifi T N, Emamie A, Rafiee N, Pashangzadeh S, Walia E, Martin NT, et al. Reovirus oncolysis and the next frontiers for this unique oncoviral immunotherapy. Semin Immunol. 2025. 10.1016/j.smim.2025.101995. 41033113

[117] Du P, Na W, Zhong J, Zhang L. Advances in preclinical and clinical studies of oncolytic virus combination therapy. Front Oncol. 2025;February:1–14. 10.3389/fonc.2025.1545542. PMC11842258 39990685

[118] Sato-dahlman M, Larocca CJ, Yanagiba C, Yamamoto M. Adenovirus and immunotherapy: advancing cancer treatment by combination. Cancers (Basel). 2020. 10.3390/cancers12051295. PMC7281656 32455560

[119] O. V Matveeva, Z. S. Guo, V. M. Senin, A. V Senina, S. A. Shabalina, and P. M. Chumakov, “Oncolysis by paramyxoviruses : preclinical and clinical studies,” *Mol. Ther. oncolytics*, no. August, pp. 1–14, 2015, 10.1038/mto.2015.17. PMC4667943 26640815

[120] Zaher KA, Alrahimi JS, Basingab FS. Newcastle disease virus virotherapy: unveiling oncolytic efficacy and immunomodulation. Biomedicines. 2024. 10.3390/biomedicines12071497. PMC11274839 39062070

[121] E. Msaouel, P., Iankov, I. D., Dispenzieri, A., & Galanis, “Attenuated oncolytic Measles Virus strains as cancer therapeutics,” *Curr. Pharm. Biotechnol.*, vol. 13, no. 9, pp. 1732–1741, 2013. 10.2174/138920112800958896 PMC3298624 21740361

[122] Guillerme J, Gregoire M. Antitumor virotherapy by attenuated measles virus (MV). Biology. 2013. 10.3390/biology2020587. PMC3960896 24832799

[123] E. Msaouel, P., Dispenzieri, A., & Galanis, “Clinical testing of engineered oncolytic measles virus strains in the treatment of cancer: An overview,” *Curr. Opin. Mol. Ther.*, vol. 11, no. 1, pp. 43–53, 2009. PMC2717625 19169959

[124] Sun J, Wang J, Xiao M, Chen L. Research progress on recombinant NDV in cancer therapy. Front Immunol. 2025;December:1–16. 10.3389/fimmu.2025.1735440. PMC12753920 41479917

[125] B. Burman, G. Pesci, and D. Zamarin, “Newcastle Disease Virus at the Forefront of Cancer Immunotherapy,” *Cancers (Basel).*, pp. 1–15, 2020. 10.3390/cancers12123552 PMC7761210 33260685

[126] J. R. Jing, Y., Bejarano, M. T., Zaias, J., & Merchan, “In vivo antimetastatic effects of uPAR retargeted measles virus in syngeneic and xenograft models of mammary cancer,” *Breast Cancer Res. Treat.*, vol. 149, no. 1, pp. 99–108, 2015, 10.1007/s10549-014-3236-8.In. PMC4302411 25519042

[127] Robinson S, Galanis E, Clinic M. Potential and clinical translation of oncolytic measles viruses. Expert Opin Biol Ther. 2018;17(3):353–63. 10.1080/14712598.2017.1288713.Potential. PMC5381660 28129716

[128] Wu J, et al. Strategic advances in targeted delivery carriers for therapeutic cancer vaccines. Int J Mol Sci. 2025. 10.3390/ijms26146879. PMC12295341 40725126

[129] Hemminki O, Manuel J, Hemminki A. Oncolytic viruses for cancer immunotherapy. J Hematol Oncol. 2020;1:1–15. 10.1186/s13045-020-00922-1 PMC7325106 32600470

[130] Maroun J, Muñoz-alía M, Ammayappan A, Schulze A, Peng K, Russell S. Designing and building oncolytic viruses. Future Virol. 2017;12:193–213. 10.2217/fvl-2016-0129 PMC5779534 29387140

[131] Kaufman HL, Shalhout SZ, Iodice G. Talimogene laherparepvec : moving from first-in-class to best-in-class. Front Mol Biosci. 2022;9(February):1–10. 10.3389/fmolb.2022.834841. PMC8901478 35274007

[132] Arellano M. Clinical uses of GM-CSF, a critical appraisal and update. Biologics. 2008;2(1):13–27. 10.2147/btt.s1355 PMC2727781 19707424

[133] Yan W, et al. Oncolytic vaccinia virus armed with GM-CSF and IL-7 enhances antitumor immunity in pancreatic cancer. Biomedicines. 2025. 10.3390/biomedicines13040882. PMC12024586 40299475

[134] Y. Xuan et al., “GM-CSF and IL-21-armed oncolytic vaccinia virus signi fi cantly enhances anti- tumor activity and synergizes with anti-PD1 immunotherapy in pancreatic cancer,” *Front. Immunol.*, no. January, pp. 1–15, 2025, 10.3389/fimmu.2024.1506632. PMC11739091 39830516

[135] J. Ajina, A., & Maher, “Prospects for combined use of oncolytic viruses and CAR T-cells,” *J. Immunother. cancer*, pp. 1–27, 2017, 10.1186/s40425-017-0294-6. PMC5696728 29157300

[136] Leparc L, Wakimoto H. Engineered retargeting to overcome systemic delivery challenges in oncolytic adenoviral therapy. Mol Ther Oncolytics. 2025;33(2):201005. 10.1016/j.omton.2025.201005. PMC12179592 40546315

[137] Alfonso L, et al. Albumin-binding adenoviruses circumvent pre-existing neutralizing antibodies upon systemic delivery. J Control Release. 2016;237:78–88. 10.1016/j.jconrel.2016.07.004. 27388756

[138] Egorova T, Starikova A. Emerging technologies tackling adeno-associated viruses (AAV) immunogenicity in gene therapy applications. Pharmaceutics. 2025. 10.3390/pharmaceutics17111492. PMC12655054 41304828

[139] Ma N, Gao J, Pang X, Wu K, Yang S, Wei H. Formulation-optimized oncolytic viruses: advancing systemic delivery and immune amplification. J Control Release. 2025. 10.1016/j.jconrel.2025.113822. 40348130

[140] Oklu R, Zhou Y, Egan JB, Duda DG, Borad MJ. Oncolytic virus delivery: from nano- pharmacodynamics to enhanced oncolytic effect. Oncolytic Virother. 2017. 10.2147/OV.S145262. PMC5687448 29184854

[141] X. Zhang, J. He, and Y. Shao, “Research progress and development potential of oncolytic vaccinia virus,” *Chin. Med. J. (Engl).*, vol. 0, no. 7, 2025. 10.1097/CM9.0000000000003585 PMC11970828 40097373

[142] Ferrucci PF, Pala L, Conforti F, Cocorocchio E. Talimogene laherparepvec (T-VEC): an intralesional cancer immunotherapy for advanced melanoma. Cancers (Basel). 2021;13:1–14. 10.3390/cancers13061383 PMC8003308 33803762

[143] Zhang T, et al. Talimogene Laherparepvec (T-VEC): a review of the recent advances in cancer therapy. J Clin Med. 2023. 10.3390/jcm12031098. PMC9917711 36769745

[144] Donoghue CO, Doepker MP, Zager JS. Talimogene laherparepvec: overview, combination therapy and current practices. Melanoma Manag. 2016;3:267–72. 10.2217/mmt-2016-0021 PMC6094674 30190898

[145] Hu JCC, et al. A phase I study of OncoVEXGM-CSF, a second-generation oncolytic herpes simplex virus expressing granulocyte macrophage colony-stimulating factor. Clin Cancer Res. 2006;12(22):6737–47. 10.1158/1078-0432.CCR-06-0759. 17121894

[146] Koch MS, Lawler SE, Chiocca EA. HSV-1 oncolytic viruses from bench to bedside: an overview of current clinical trials. Cancers (Basel). 2020. 10.3390/cancers12123514. PMC7760226 33255871

[147] M. I. Dummer, R., Gyorki, D. E., Hyngstrom, J., Berger, A., Conry, R., Demidov, L., Sharma, A., Treichel, S. A., Gorski, K., Anderson, A., Faries, M., & Ross, “Primary 2-year (yr) results of a phase II, multicenter, randomized, open-label trial of efficacy and safety for talimogene laherparepvec (T-VEC) neoadjuvant (neo) treatment (tx) plus surgery (surg) vs surg in patients (pts) with resectable stage IIIB-IVM1,” *Ann. Oncol.*, vol. 30, 2019, 10.1093/annonc/mdz394.063.

[148] J. An et al., “Single-cell analysis of oncolytic virotherapy in adult T-cell leukemia/lymphoma,” *J. Immunother. cancer*, pp. 1–6, 2025, 10.1136/jitc-2024-011265. PMC11938254 40132906

[149] Pol J, Kroemer G, Galluzzi L. First oncolytic virus approved for melanoma immunotherapy. Oncoimmunology. 2016. 10.1080/2162402X.2015.1115641. PMC4760283 26942095

[150] Hu JCC, et al. Cancer Therapy : Clinical A Phase I Study of OncoVEX GM-CSF, a Second-Generation Oncolytic Herpes Simplex Virus Expressing Granulocyte Macrophage Colony-Stimulating Factor. Cancer Ther Clin. 2006;5(22):6737–47. 10.1158/1078-0432.CCR-06-0759. 17121894

[151] Senzer NN, et al. Phase II clinical trial of a granulocyte-macrophage colony-stimulating factor – encoding, second-generation oncolytic herpesvirus in patients with unresectable metastatic melanoma. J Clin Oncol. 2015;27(34):5763–71. 10.1200/JCO.2009.24.3675. 19884534

[152] Kaufman HL, Kim DW, Deraffele G, Mitcham J, Coffin RS, Kim-schulze S. Local and distant immunity induced by intralesional vaccination with an oncolytic herpes virus encoding GM-CSF in patients with stage IIIc and IV melanoma. Ann Surg Oncol. 2010;50:718–30. 10.1245/s10434-009-0809-6. 19915919

[153] Kaufman HL, Bines SD. OPTIM trial: a phase III trial of an oncolytic herpes virus encoding GM-CSF for unresectable stage III or IV melanoma. Future Oncol. 2010. 10.2217/fon.10.66. 20528232

[154] Andtbacka RHI, et al. Talimogene laherparepvec improves durable response rate in patients with advanced melanoma. J Clin Oncol. 2015;33(25):2780–8. 10.1200/JCO.2014.58.3377. 26014293

[155] J. M. Ressler et al., “Real-life use of talimogene laherparepvec (T-VEC) in melanoma patients in centers in Austria, Switzerland and Germany,” *J. Immunother. cancer*, pp. 1–9, 2021, 10.1136/jitc-2020-001701. PMC7898852 33608376

[156] M. M. Yamada et al., “Real-world outcomes of talimogene laherparepvec as salvage therapy for advanced melanoma,” *J. Immunother. cancer*, pp. 1–9, 2025, 10.1136/jitc-2025-013564. PMC12742145 41448830

[157] M. Robilotti, E., & Kamboj, “Infections in Patients Receiving TVEC Therapy,” *Open Forum Infect. Dis.*, vol. 6, 2019, 10.1093/ofid/ofz360.1976.

[158] C. Y. Bayan et al., “The Role of Oncolytic Viruses in the Treatment of Melanoma,” *Curr. Oncol. Rep.*, vol. 20, no. 10, 2019, 10.1007/s11912-018-0729-3.The. PMC6450085 30145781

[159] Doni S, Str I, Proboka G, Jonsson B, Venskus D, Muceniece A. Adapted ECHO-7 virus Rigvir immunotherapy (oncolytic virotherapy) prolongs survival in melanoma patients after surgical excision of the tumour in a retrospective study. Melanoma Res. 2011;25(December):421–6. 10.1097/CMR.0000000000000180. PMC4560272 26193376

[160] Alberts P, Tilgase A, Rasa A, Venskus D. The advent of oncolytic virotherapy in oncology: the Rigvir ® story. Eur J Pharmacol. 2018;837(August):117–26. 10.1016/j.ejphar.2018.08.042. 30179611

[161] K. Lundstrom and K. Lundstrom, “New frontiers in oncolytic viruses : optimizing and selecting for virus strains with improved efficacy New frontiers in oncolytic viruses : optimizing and selecting for virus strains with improved efficacy,” *Biol. Targets Ther.*, vol. 5475, 2018, 10.2147/BTT.S140114. PMC5810530 29445265

[162] Mineta RLMT, Rabkin SD, Yazaki T, Hunter WD. Attenuated multi–mutated herpes simplex virus–1 for the treatment of malignant gliomas. Nat Med. 1995;1(9):938–43. 10.1038/nm0995-938 7585221

[163] Todo T, Martuza RL, Rabkin SD, Johnson PA. Oncolytic herpes simplex virus vector with enhanced MHC class I presentation and tumor cell killing. Proc Natl Acad Sci U S A. 2001;98(11):6396–401. 10.1073/pnas.101136398. PMC33479 11353831

[164] Ino Y, Todo T. Clinical development of a third-generation oncolytic HSV-1 (G47∆) for malignant glioma. Gene Ther Regul. 2010;5(1):101–11. 10.1142/S1568558610000185.

[165] Fukuhara H, Ino Y, Todo T. Oncolytic virus therapy: a new era of cancer treatment at dawn. Cancer Sci. 2016. 10.1111/cas.13027. PMC5084676 27486853

[166] S. Taguchi, H. Fukuhara, and T. Todo, “Oncolytic virus therapy in Japan: progress in clinical trials and future perspectives,” *Jpn. J. Clin. Oncol.*, pp. 1–9, 2018, 10.1093/jjco/hyy170. 30462296

[167] Feng Y, et al. Immune checkpoint inhibitors combined with oncolytic virotherapy: synergy, heterogeneity, and safety in cancer treatment. Oncol Res. 2025;33(12):3801–36. 10.32604/or.2025.067824. PMC12712690 41425703

[168] Chesney JA, et al. Talimogene laherparepvec in combination with ipilimumab versus ipilimumab alone for advanced melanoma : 5- ­ year final analysis of a phase II trial. J Immunother cancer. 2023;11(5):1–9. 10.1136/jitc-2022-006270. PMC10163510 37142291

[169] Chesney JA, Ribas A, Long GV, Kirkwood JM, Dummer R. Placebo-controlled, global phase III trial of talimogene laherparepvec combined with pembrolizumab for advanced melanoma. J Clin Oncol. 2023;41(3):528–40. 10.1200/JCO.22.00343. PMC9870217 35998300

[170] S. H.-L. et Al, “Abstract 3275: Combination of talimogene laherparepvec (T-VEC) with pembrolizumab (pembro) in advanced melanoma patients following progression on a prior PD-1 inhibitor: SWOG S1607,” *Cancer Res.*, vol. 83, no. 7, 2023.

[171] Zijlker LP, et al. Neoadjuvant T-VEC + nivolumab combination therapy for resectable early metastatic ( stage IIIB / C / D-IV M1a ) melanoma with injectable disease : NIVEC trial. J Clin Oncol. 2023;11:9546. 10.1200/JCO.2023.41.16. PMC9351098 35927710

[172] V. M. Neil and S. W. Lee, “Advancing Cancer Treatment: A Review of Immune Checkpoint Inhibitors and Combination Strategies,” *Cancers (Basel).*, pp. 1–32, 2025. 10.3390/cancers17091408 PMC12071127 40361336

[173] Nguyen VP, et al. A pilot study of neoadjuvant nivolumab, ipilimumab, and intralesional oncolytic virotherapy for HER2-negative breast cancer. Cancer Res Commun. 2023;3(August):1628–37. 10.1158/2767-9764.CRC-23-0145. PMC10445661 37621406

[174] Z. Qian, Q. Gao, and W. Zhang, “Combination of oncolytic viruses and immune checkpoint inhibitors for treatment of high-grade gliomas,” *Front. Neurol.*, no. February, 2026, 10.3389/fneur.2026.1683726. PMC12932173 41756179

[175] C. M. Kelly et al., “A phase II study of talimogene laherparepvec (T-VEC) and pembrolizumab in patients with advanced sarcoma: Results of expansion cohorts.,” *J. Clin. Oncol.*, vol. 41, no. 16_suppl, p. 11570, 2023, 10.1200/JCO.2023.41.16_suppl.11570.

[176] Chen F, et al. SMAC-armed oncolytic virotherapy enhances the anticancer activity of PD1 blockade by modulating PANoptosis. Biomark Res. 2025;13(1):8. 10.1186/s40364-025-00726-w. PMC11721257 39789615

[177] El-Sayes N, Vito A, Salem O, Workenhe ST, Wan Y, Mossman K. A combination of chemotherapy and oncolytic virotherapy sensitizes colorectal adenocarcinoma to immune checkpoint inhibitors in a cDC1-dependent manner. Int J Mol Sci. 2022;23(3):1754. 10.3390/ijms23031754. PMC8915181 35163675

[178] Huppert LA, et al. Phase Ib study of talimogene laherparepvec (T-VEC) in combination with chemotherapy (CT) or endocrine therapy (ET) in patients with metastatic, unresectable, or locoregionally recurrent HER2-negative breast cancer (BC). J Clin Oncol. 2023;41(16_suppl):1091. 10.1200/JCO.2023.41.16_suppl.1091.

[179] Monga V, et al. Intratumoral talimogene laherparepvec injection with concurrent preoperative radiation in patients with locally advanced soft-tissue sarcoma of the trunk and extremities: phase IB/II trial. J Immunother Cancer. 2021;9(7):e003119. 10.1136/jitc-2021-003119. PMC8327848 34330766

[180] E. Ponterio, T. L. Haas, and R. De Maria, “Oncolytic virus and CAR-T cell therapy in solid tumors,” *Front. Immunol.*, vol. 15, 2024, 10.3389/fimmu.2024.1455163. PMC11557337 39539554

[181] Y. Liu et al., “Oncolytic herpes simplex virus delivery of dual CAR targets of CD19 and BCMA as well as immunomodulators to enhance therapeutic efficacy in solid tumors combined with CAR T cell therapy,” *Front. Oncol.*, vol. 12, 2022, 10.3389/fonc.2022.1037934. PMC9638445 36353540

[182] Y. Zheng et al., “Abstract 5072: ‘Armed’ oncolytic herpes simplex virus enables CD19 CAR-T for solid tumor cell treatment as a combination therapy,” *Cancer Res.*, vol. 83, no. 7_Supplement, p. 5072, 2023, 10.1158/1538-7445.AM2023-5072.

[183] S. Jin et al., “Oncolytic Therapy: Delivery System and New Therapeutic Strategies for Cancer,” *MedComm*, vol. 7, no. 4, 2026, 10.1002/mco2.70725. PMC13066727 41970230

[184] Liang Y, et al. Systemic delivery of glycosylated-PEG-masked oncolytic virus enhances targeting of antitumor immuno-virotherapy and modulates T and NK cell infiltration. Theranostics. 2023;13(15):5452–68. 10.7150/thno.87498. PMC10614686 37908722

[185] Wang X, et al. Overcoming resistance in oncolytic virotherapy: Nano-engineered solutions for systemic delivery and efficacy boost. Drug Resist Updat. 2026;85:101345. 10.1016/j.drup.2025.101345. 41485410

[186] Wang J, et al. IRF7 drives resistance to oncolytic virotherapy by restricting viral replication and suppressing antitumor immunity. Biochem Biophys Res Commun. 2025;782:152605. 10.1016/j.bbrc.2025.152605. 40925101

[187] Robilotti E, Kamboj M. 2298. Infections in patients receiving TVEC therapy. Open Forum Infect Dis. 2019;6(Supplement_2):S787–8. 10.1093/ofid/ofz360.1976.

[188] Larrieux A, Sanjuán R. Murine colon cancer derived cells exhibit heterogeneous resistance profiles against an oncolytic virus. Sci Rep. 2024;14(1):27209. 10.1038/s41598-024-78313-6. PMC11549347 39516525

[189] Neulinger-Muñoz M, et al. Human retrotransposons and the global shutdown of homeostatic innate immunity by oncolytic parvovirus H-1PV in pancreatic cancer. Viruses. 2021;13(6):1019. 10.3390/v13061019. PMC8228339 34071585

[190] Göbel S, et al. Characterization of a quail suspension cell line for production of a fusogenic oncolytic virus. Biotechnol Bioeng. 2023;120(11):3335–46. 10.1002/bit.28530. 37584190

[191] van der Loo JCM, Wright JF. Progress and challenges in viral vector manufacturing. Hum Mol Genet. 2016;25(R1):R42–52. 10.1093/hmg/ddv451. PMC4802372 26519140

[192] J.-W. Wang, Q. Feng, J.-H. Liu, and J.-J. Xun, “Opportunities, challenges, and future perspectives of oncolytic virus therapy for malignant melanoma,” *Front. Immunol.*, vol. 16, 2025, 10.3389/fimmu.2025.1653683. PMC12443682 40977706

[193] Xiao D, Zhang H, Liu Y, Li Y, Li G, Ning Y. Oncolytic viruses: advanced strategies in cancer therapy. Signal Transduct Target Ther. 2026;11(1):45. 10.1038/s41392-025-02343-3. PMC12873401 41639062

[194] Fretwell EC, Houldsworth A. Oncolytic virus therapy in a new era of immunotherapy, enhanced by combination with existing anticancer therapies: turn up the heat! J Cancer. 2025. 10.7150/jca.102285. PMC11905400 40092697

[195] Muthukutty P, Yoo SY. Oncolytic virus engineering and utilizations: cancer immunotherapy perspective. Viruses. 2023. 10.3390/v15081645. PMC10459766 37631987

[196] T. Azad et al., “Synthetic virology approaches to improve the safety and ef fi cacy of oncolytic virus therapies,” *Nat. Commun.*, pp. 1–18, 2023, 10.1038/s41467-023-38651-x. PMC10213590 37236967

[197] Xiao H, et al. Oncolytic HSV-1 expressing GM-CSF and IL-12 enhances anti-tumor efficacy in immunocompetent murine melanoma model. Virol Sin. 2025;40(6):977–88. 10.1016/j.virs.2025.11.002. PMC12826977 41232605

[198] Z. Wang, P. Sun, Z. Li, and S. Xiao, “Clinical Advances and Future Directions of Oncolytic Virotherapy for Head and Neck Cancer,” *Cancers (Basel).*, pp. 1–14, 2023. 10.3390/cancers15215291 PMC10650136 37958464

[199] Li L, Liu S, Han D, Tang B, Ma J. Delivery and biosafety of oncolytic virotherapy. Front Oncol. 2020;10(April):1–15. 10.3389/fonc.2020.00475. PMC7176816 32373515

[200] F. Prestwich, R. J., Harrington, K. J., Pandha, H. S., Vile, R. G., Melcher, A. A., & Errington, “Oncolytic viruses : a novel form of immunotherapy,” *Expert Rev. Anticancer Ther.*, vol. 8, no. 10, pp. 1581–1588, 2009, 10.1586/14737140.8.10.1581.Oncolytic. PMC2729453 18925850

[201] El-tanani M, Rabbani SA, Patni MA, Babiker R. Efficacy, safety and predictive biomarkers of oncolytic virus therapy in solid tumors: a systematic review and meta-analysis. Vaccines (Basel). 2025. 10.3390/vaccines13101070. PMC12567567 41150456

[202] Omolekan TO, et al. Viral warfare: unleashing engineered oncolytic viruses to outsmart cancer’s defenses. Front Immunol. 2025. 10.3389/fimmu.2025.1618751. PMC12414947 40927720

[203] H. Singh, I. Mijakovic, P. Singh, and C. De Marco, “Advances in precision oncology using patient-derived organoids and functional biomaterials,” *Front. cell Dev. Biol.*, no. September, pp. 1–28, 2025, 10.3389/fcell.2025.1670328. PMC12518345 41098309

[204] Liu Y, Wu W, Cai C, Zhang H, Shen H, Han Y. Patient-derived xenograft models in cancer therapy: technologies and applications. Sig Transduct Target Ther. 2022. 10.1038/s41392-023-01419-2. PMC10097874 37045827

[205] Menotti L, Vannini A. Oncolytic viruses in the era of omics, computational technologies, and modeling: thesis, antithesis, and synthesis. Int J Mol Sci. 2023. 10.3390/ijms242417378. PMC10743452 38139207

[206] Appleton E, Chiocca EA, Ungerechts G, Melcher A, Vile R. Oncolytic viruses as anticancer agents: clinical progress and remaining challenges. Lancet. 2025;406(10509):1295–312. 10.1016/S0140-6736(25)01206-1. PMC13625015 40975614

[207] Y. R. Murciano-goroff, A. B. Warner, and J. D. Wolchok, “The future of cancer immunotherapy : microenvironment- targeting combinations,” *Cell Res.*, no. May, 2020, 10.1038/s41422-020-0337-2. PMC7264181 32467593

[208] Wong HH, Lemoine NR, Wang Y. Oncolytic viruses for cancer therapy: overcoming the obstacles. Viruses. 2010. 10.3390/v2010078. PMC2883714 20543907

